# Phage Cocktail Development for Bacteriophage Therapy: Toward Improving Spectrum of Activity Breadth and Depth

**DOI:** 10.3390/ph14101019

**Published:** 2021-10-03

**Authors:** Stephen T. Abedon, Katarzyna M. Danis-Wlodarczyk, Daniel J. Wozniak

**Affiliations:** 1 Department of Microbiology, The Ohio State University, Columbus, OH 43210, USA; daniel.wozniak@osumc.edu; 2 Department of Microbial Infection and Immunity, The Ohio State University, Columbus, OH 43210, USA; danis-wlodarczyk.1@osu.edu

**Keywords:** antimicrobial resistance, bacteriophage therapy, combating resistance, combination therapy, host range, JavaScript, online apps, quantitative biology

## Abstract

Phage therapy is the use of bacterial viruses as antibacterial agents. A primary consideration for commercial development of phages for phage therapy is the number of different bacterial strains that are successfully targeted, as this defines the breadth of a phage cocktail’s spectrum of activity. Alternatively, phage cocktails may be used to reduce the potential for bacteria to evolve phage resistance. This, as we consider here, is in part a function of a cocktail’s ‘depth’ of activity. Improved cocktail depth is achieved through inclusion of at least two phages able to infect a single bacterial strain, especially two phages against which bacterial mutation to cross resistance is relatively rare. Here, we consider the breadth of activity of phage cocktails while taking both depth of activity and bacterial mutation to cross resistance into account. This is done by building on familiar algorithms normally used for determination solely of phage cocktail breadth of activity. We show in particular how phage cocktails for phage therapy may be rationally designed toward enhancing the number of bacteria impacted while also reducing the potential for a subset of those bacteria to evolve phage resistance, all as based on previously determined phage properties.

## 1. Introduction

“...a characteristic that is often touted as a benefit—that of the exquisite selectivity of phage, which reduces the ‘off-target’ effects on commensal bacteria—also conveys a major challenge for its practical use, namely the ability to ensure effectiveness across disparate isolates of the same pathogen. ...the use of multi-component cocktails that are screened against large banks of pathogen or careful engineering of phage to increase the phage’s host range may be needed to circumvent this issue. Intimately connected to this issue is the reality of resistance to phage by the pathogen, an inevitable consequence of their co-evolutionary relationship. It is therefore likely that resistance development and characterization of this process will be among the chief regulatory concerns. To address the problem of resistance, strategic approaches in selecting phage may be needed...”—Randal Kincaid [1].

Bacteriophages, or phages—the viruses of bacteria—can be used as antibacterial agents against numerous types of bacterial infections. In this modern era, this is especially directed toward treating chronic bacterial infections against which antibiotic treatments either have been unsuccessful or are otherwise not possible [2,3,4,5,6]. Such phage therapy can involve application of only a single phage type (monophage). The potential for a monophage to control a given bacterial infection, however, is in part a function of the breadth of that phage’s host range [7,8,9,10], which is equivalent pharmacologically to the breadth of the spectrum of activity of a chemical antibacterial agent [11]. One general means of enhancing spectrum of activity breadth beyond what is achievable by a monophage is to combine treatments. Phages, for example, can be combined with non-phage agents such as antibiotics [12,13,14,15,16,17,18,19,20,21], or phages can instead be combined with other phages possessing different host ranges. The latter, phage–phage combination therapies, can be described as polyphage treatments. More commonly, these are known as phage cocktails [22,23,24,25].

Phage cocktail spectrum of activity may be optimized in different ways. Cocktails, perhaps most familiarly, can be developed to target a diversity of bacterial types. Alternatively, phage cocktails may be designed to inhibit the potential for bacteria to evolve phage resistance. These distinctions we refer to, respectively, as being functions of a cocktail’s *breadth* of activity vs. a cocktail’s *depth* of activity. Ideally, all phage cocktails would display both substantial breadth and substantial depth, but for different purposes cocktail breadth of activity rather than cocktail depth of activity might be emphasized, e.g., Haines et al. [26], or instead vice versa. Related to depth of activity is the potential for single bacterial mutations to result in the blocking of the antibacterial action of two or more different phage types, i.e., the occurrence of cross resistance [27,28,29,30].

Here, we describe the different ways that phage cocktails may be quantitatively assessed in light especially of their breadth of activity. We present three algorithms, the first two of which are based on previously obtained phage host-range data while the third depends as well on determination of the potential for bacteria to mutate to cross resistance to different phages. The first is a recognizable calculation of a cocktail’s activity breadth, i.e., number of bacteria affected. The second is an equivalent analysis but which takes into account a cocktail’s depth of activity as well, i.e., determination of a cocktail’s breadth of activity in light of this important aspect of the cocktail’s potential to also limit bacterial evolution of phage resistance. The third is a determination of cocktail breadth of activity, again in light of cocktail depth of activity, but while also taking into account the potential for bacteria to mutate to cross resistance to different phages. We have also developed two online apps toward automating these analyses [31,32] (see Supplementary Materials for archived code).

Together, these approaches allow first for a rapid assessment of proposed phage cocktails in terms of their potential to impact a diversity of possible bacterial targets. Second, they provide an assessment of what fraction of those targeted bacteria—generally somewhat less than 100%—a cocktail can impact with more than one type of its constituent phages, as toward limiting bacterial evolution of phage resistance. We start, however, by considering the functional diversity that can be found among typical phage cocktails, particularly from the perspectives of both cocktail spectrum of activity breadth and cocktail spectrum of activity depth. 

## 2. Cocktails Can Emphasize Different Spectra of Activity

**Figure 1:**
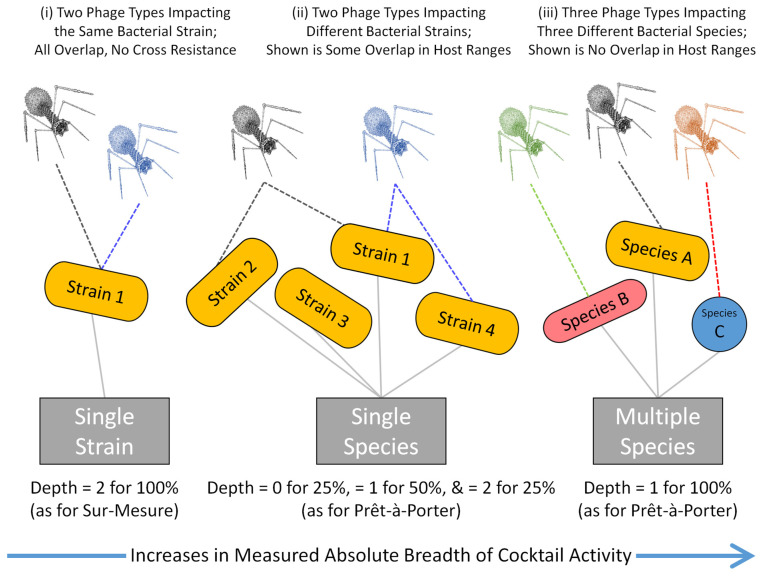
Variations in phage cocktail breadth and depth of activity. Phage cocktails can be designed to impact multiple species (**right**), multiple strains of a single species (**middle**), or just a single bacterial strain (**left**). Our emphasis here primarily is toward combining the properties of the middle category with those of the leftward category. “Sur-mesure” means that a cocktail is custom-made or personalized; “Prêt-à-porter” is translated literally as ‘ready-to-wear’, but meaning here that it is not custom made for a specific patient. Spectrum of activity breadth can be considered in absolute terms, e.g., impacting one species versus impacting three species (see the arrow at the bottom of the figure). Spectrum of activity breadth can also be considered in relative terms, e.g., impacting 75% rather than 100% of the strains making up a single bacterial species. This latter concept we describe below as “Breadth_1_”, read as “Breadth subscript 1”. Spectrum of activity depth refers to how many phages target a single, specific bacterial strain, especially given an inability of that bacterial strain to mutate to cross resistance to the different phages. To the left, the cocktail targets a single bacterial strain, designated as “Strain 1” in the figure, with a depth of 2. This means that Strain 1 is being targeted by two different phages, especially two phages that are found in two different cross-resistance group. As this is the only strain shown to the left, and therefore is 100% of bacterial strains shown there, in the figure this is indicated as “Depth = 2 for 100%”. In the middle, with the cocktail targeting multiple strains of a single bacterial species, relative breadth of activity is 75% ( = percent strains with Depth ≥ 1), as impacting “Strain 1” (Depth = 2), “Strain 2” (Depth = 1), and “Strain 4” (Depth = 1), but not “Strain 3” (Depth = 0). Thus, for one-quarter of the strains the depth is equal to 0 (“Depth = 0 for 25%”), for one-half depth instead is equal to 1 (Depth “= 1 for 50%”), and for another one-quarter depth is equal to 2 (Depth “= 2 for 25%”). To the right, the cocktail is impacting three different bacterial species, A, B, and C, using three different phages. Relative breadth of activity there is 100% (percentage of shown strains targeted with Depth ≥ 1), but depth of activity is only 1 for each of those strains (“Depth = 1 for 100%”).

In this section we consider how phage cocktails can be developed toward impacting qualitatively different categories of bacterial targets, as summarized in Figure 1. Our emphasis is on the host ranges of cocktail-constituent phages along with resulting differences in cocktail spectrum of activity breadth vs. depth.

### 2.1. Emphasizing Breadth vs. Depth of Activity

We start with the targeting of multiple bacterial species, i.e., “Multiple species targets” (Figure 1, right). These phage cocktails are available commercially, especially as produced in former republics of the Soviet Union. Though by design these cocktails supply ample breadth of activity, i.e., to include more than one bacterial species, their development is not our emphasis here. We then consider “Single species targets” (Figure 1, middle), which typically are envisaged as commercial products developed for North American and European markets. These also tend to emphasize breadth of activity, though they do not need to have as great a breadth of activity as cocktails with multiple species targets. It is the development of cocktails that target single species that is our emphasis here. Lastly, we consider cocktails with “Single strain targets” (Figure 1, left). The latter may be especially used to combat bacterial evolution of phage resistance, but to do so they need to prioritize enhancement of cocktail depth of activity and not necessarily cocktail breadth of activity as well. It is addressing the challenge of combining both spectrum of activity breadth and spectrum of activity depth within individual, commercially available phage cocktails that is our particular emphasis here.

Other phage and cocktail properties are also relevant to phage use in therapy. These include virion stability (desirable for phage therapy), encoding of bacterial virulence factors (undesirable for phage therapy), and propensity to generate bacterial lysogens (also undesirable for phage therapy) [24,33,34,35]. Furthermore, there are issues around whether phages should be supplied in serial (one monophage type at a time) rather than in parallel (as cocktails), whether upon infecting the same bacterium individual phages will interfere with each other’s ability to produce new virions, whether resistance to individual phages will or will not readily evolve in vivo vs. in vitro, and whether bacterial mutations to phage resistance can result in reduced bacterial fitness or virulence, e.g., [22,28,29,36,37,38,39,40,41,42]. These additional considerations lie outside of the scope of this piece, however. 

### 2.2. Multiple Species Targets

Phage cocktails can consist of individual phages which in aggregate can impact different bacterial species, e.g., bacterial species ‘A’ and bacterial species ‘B’ (Figure 1, right) [24,43]. Such cocktails, targeting multiple species, tend to be used empirically to treat general disease states. For example, skin or soft-tissue infections can be caused by different species of bacteria and can be treated using the phage cocktail known as Pyophage [44,45]. Pyophage-type cocktails can in fact target multiple bacterial genera, such as *Enterococcus*, *Escherichia*, *Proteus*, *Pseudomonas*, *Staphylococcus*, and *Streptococcus*.

Given the typically limited host range of phages [7,8], to achieve this broad spectrum of activity a cocktail would tend to consist of at least one phage that targets species A and at least one that targets species B (Figure 1, right), etc. Alternatively, such a very broad spectrum of activity might be achievable with fewer phages if individual phages were able to infect multiple bacterial species [8,46], so-called polyvalence [47,48]. However, the more diverse the bacteria targeted, e.g., Gram positive vs. Gram negative, the less likely it is that appropriate polyvalent phages may be isolated. Though by definition such multiple-species cocktails would display considerable breadth of activity, multiple-species cocktails, as noted, are not our emphasis here.

### 2.3. Single Species Target: Emphasizing Cocktail Breadth of Activity

Cocktails targeting a single bacterial species ideally will contain phages with host ranges that, on aggregate, impact a large fraction of the bacterial strains making up that species. The primary reason for needing such cocktails, rather than therapies based on individual monophages, is that phages in a majority of cases possess host ranges that fall far short of encompassing the entirety of a bacterial species [24,47], though such narrow host ranges are not always the case [49,50]. To achieve broad activity spectra for phage therapy, it therefore often becomes necessary to combine together phages possessing divergent host ranges. Commercially available phage formulations designed for empirical antibacterial use typically will take this form. Such cocktails can be described as ‘prêt-à-porter’, idiomatically meaning ‘off the shelf’ [51], and assisting the development of prêt-à-porter phage cocktails, in terms of their spectra of activity, is our primary emphasis here. 

Single-species cocktails should consist, at a minimum, of one phage that targets one group of bacterial strains and a second phage targeting a somewhat different group of bacterial strains, with both groups of bacterial strains associated with the same species (Figure 1, middle). It is fairly typical in the phage therapy literature for researchers to employ such cocktails; ones that have been designed to target a single bacterial species more broadly than individual constituent phages are capable, and a characteristic sign that this is the case is when tables of phage host range data are used to justify the inclusion of specific phages in resulting cocktails. We describe these cocktails, even though they are targeting only a single rather than multiple bacterial species, as generally emphasizing spectrum of activity *breadth* in their design, rather than necessarily emphasizing cocktail *depth* of activity as well.

### 2.4. Single Strain Target: Emphasizing Depth of Activity

A phage cocktail can also be formulated to explicitly contain more than one phage type that targets a single bacterial strain. Such cocktails may be used when a bacterial pathogen has been isolated from an individual patient, or may instead be used to target a specific pathogenic bacterial strain that is circulating within a community. This approach potentially results in greater depth of activity against the targeted strain (Figure 1, left) than a cocktail that has been formulated instead with emphasis on targeting multiple bacterial strains (Figure 1, middle).

As cocktails optimized to enhance depth of activity would be employed especially to combat bacterial evolution of phage resistance, different phages making up such a cocktail should target different resistance genotypes that could evolve in the course of phage treatment. These often would be bacterial mutants defective in phage receptors [52]. More generally, enhancement of a phage cocktail’s depth of activity toward interfering with bacterial evolution of phage resistance will depend on a low potential by target bacteria to mutate to cross resistance to the different phages making up a cocktail or, as Regeimbal et al. [53] make this same point (p. 5807), “as distinct a host range for strains of the targeted pathogen as possible. Combining such phages into a single treatment cocktail may extend the utility of a personalized cocktail and reduce the frequency of phage cocktail resistance and therapeutic failure”.

### 2.5. Sur-Mesure vs. Prêt-à-Porter Targeting

Our expectations are that prêt-à-porter polyphage products, as off-the-shelf cocktails, would not necessarily be optimized to also combat bacterial evolution of phage resistance across a reasonably large subset of the bacterial strains being targeted, unless those products have been formulated explicitly to assure such depth of activity. Specifically, prêt-à-porter cocktails will not inevitably contain more than one phage that negatively impacts each or even most of the possible bacterial strains affected, though this will be more likely if the number of targeted bacterial strains is somewhat limited and the cocktail has been designed explicitly toward that aim. Prêt-à-porter cocktails therefore may only be effective by chance at combating the evolution of phage resistance by a breadth of targeted bacterial strains.

Even independent of issues of bacterial evolution of phage resistance, a diversity of phage characteristics could have utility within a cocktail, especially given lack of full knowledge of phage antibacterial activity during treatments. More personalized, or ‘Sur-mesure’ treatments—especially cocktails that are designed to target only one or very few bacterial strains—will, on the other hand, ideally be designed particularly for the sake of reducing the potential of targeted bacteria to in fact evolve such resistance. That is, it can be useful for phages found in sur-mesure cocktails to independently target different possible resistance genotypes that can readily evolve from a single phage-targeted bacterial strain [54,55]. Such cocktails could even include phages that target phage-resistant bacterial mutants but not also the original bacterial strain [53,56]. With such sur-mesure treatments, breadth of activity should not otherwise be relevant to cocktail success. This is because no more than one or very few bacterial strains, along with their mutant daughter strains, would be explicitly targeted by the cocktail. Indeed, Regeimbal et al. [53] show that it is possible for a sur-mesure cocktail that is effective at combatting bacterial evolution of phage resistance to also display an extremely narrow breadth of antibacterial activity.

Relative to the designing of sur-mesure cocktails with their single- or few-strain specificity toward enhanced depth of activity, we have found the goal of designing phage cocktails targeting many bacterial strains—while still reducing the likelihood of bacterial evolution of phage resistance—to be challenging. Reducing that challenge, however, is the primary issue that we address here. In any case, achieving substantial cocktail depth of activity is still dependent upon finding appropriate phages, as the following section considers. See Table 1 for further comparison of cocktails targeting multiple bacterial species, a single bacterial species, or single bacterial strains as well as sur-mesure vs. prêt-à-porter targeting.

## 3. Cross Resistance

Reductions in the potential of bacteria to evolve resistance is achievable using phage cocktails, but only to the extent that bacterial acquisition of resistance to one phage does *not* result in a high probability of evolution of resistance to all of the other phages making up a cocktail. Formally, this is described as avoiding or at least minimizing the potential for bacterial evolution of cross resistance, which can be viewed as bacterial mutations having pleiotropic phage-resistance effects. The pleiotropy in the case of cross resistance would be mutation to resistance to one phage—particularly resistance to the selecting treatment phage—that results also in resistance by the same bacterium to a second or additional phages. These pleiotropic effects typically would be the result of point mutations, i.e., as directly impacting only a single gene product, rather than the result of deletion mutations that directly impact the functioning of multiple genes. Though not necessarily limited to this example, cross resistance could be a consequence of bacterial mutational loss of a phage adsorption-receptor molecule. Such cross resistance is as opposed to what Fazzino et al. [57] describe as dual resistance, which is bacterial resistance to two different phages as resulting from two different resistance mutations, one resistance mutation for each phage. Minimizing the potential for bacteria to mutate to cross resistance to the phages found within a phage cocktail can be left to chance, or ideally may instead be explicitly incorporated into cocktail development.

### 3.1. Minimizing Cross Resistance toward Improving Depth of Activity

Using phage cocktails to reduce the potential for bacteria to evolve phage resistance requires that rates of bacterial mutation to combined resistance to a polyphage (phage cocktail) be substantially reduced relative to rates of bacterial mutation to resistance to individual monophages. Ideally, these rates of mutation to resistance would be lower than the reciprocal of the number of targeted bacteria present, e.g., lower than 10^−7^ mutations per bacterium per cell division if 10^7^ bacteria are targeted. Demerec and Fano [36] measured rates of mutation by *Escherichia coli* B to phages T1, T3, T4, T5, T6, and T7, which ranged on average for a given phage type from roughly 10^−7^ to 10^−8^ mutations per bacterium per cell division. Rates of mutation by *E. coli* BW25113 to phage U136B appear to be somewhat higher, with a frequency of 10^−6^. Rates of mutation by *Pseudomonas aeruginosa* P14 to resistance to phage DMS3*vir* were found to be higher still, at nearly 10^−4^, though this may be due in part to the large mutational target of the multiple genes necessary for synthesis of the pilus this phage uses as its adsorption receptor [58]. See Silva et al. [52] for a review of phage receptor molecules.

For rates of bacterial mutation to phage resistance of 10^−4^, 10^−6^, or 10^−8^ mutations per bacterium per cell division, we would expect a bacterial population of 10^9^ individual bacteria to contain roughly one hundred thousand (10^5^), one thousand (10^3^), or ten (10^1^) bacterial cells that are phage resistant, respectively. By contrast, rates of *E. coli* B mutation for resistance to phage T2 are much lower, owing to the independent recognition by this phage of two different primary receptor molecules displayed by that host strain [59]. Other dual receptor-using phages exist as well [46,60,61,62,63]. Though presumably phages targeting two different receptor molecules for their primary bacterial-attachment process can be useful for phage therapy purposes, for individual phage-bacterium combinations dual receptor recognition does not appear to be common.

Achieving lower rates of bacterial mutation to resistance to treatment phages is more commonly accomplished by employing cocktails consisting of multiple phages that use, e.g., different bacterial receptor molecules for adsorption. This is so that bacterial mutation to cross resistance to different treatment phages is less likely to occur. Ideally, targeted bacteria would then need to acquire two mutations to attain resistance to two phages that use, e.g., two different bacterial surface molecules as adsorption receptors. If a bacterium’s mutation rate to resistance to a phage is, for example, equal to 10^−6^ mutations per bacterium per cell division, then acquiring two such mutations independently should occur at a rate per cell division of 10^−6^ × 10^−6^ = 10^−12^. The reciprocal of this latter number ideally will exceed the number of bacteria present within an infection. In actuality, however, if rare single mutations conferring cross resistance can still occur [36], then the rate of mutation to both of these example phages may be greater than 10^−12^. Nevertheless, the lower the rates of bacterial mutation to resistance to two different phage types, then the more useful a combination of those phages should be toward reducing the potential for bacterial evolution of phage resistance during phage treatments, in this example lower than 10^−6^ if not necessarily as low as 10^−12^.

Cocktail depth of activity is a function not just of the number of phages that can negatively affect an individual bacterial strain, but also the number of phages for which a targeted bacterium has relatively non-overlapping resistance strategies. Therefore, if a cocktail consists of ten phages, but cross resistance by the targeted bacterium readily occurs to nine of them, then the depth of activity would be just two for the cocktail against that bacterium. Though this would represent only two phage cross-resistance groups, such a cocktail still should reduce bacterial mutation rates to all of the phages present to somewhat less than the mutation rate to any one of those phages alone. Reductions in bacterial rates of mutation to the entire cocktail nonetheless would not be as low as would be the case were cocktail phages to fall within more than two cross-resistance groups.

Alternatively, if those first nine phages making up the same cross-resistance group were used together, excluding the one phage that is found in the alternative cross-resistance group, then rates of bacterial mutation to phage resistance instead may not be greater than the rate of mutation to resistance to a single phage alone. In other words, just because a phage cocktail contains more than one phage targeting a certain bacterial strain, that does not guarantee that rates of bacterial mutation to resistance to all of the phages found within a cocktail will be meaningfully reduced in comparison to monophage use. Achieving substantial cocktail depth of activity thus is dependent on finding appropriate combinations of phages, ones for which bacterial mutation to cross resistance is less likely.

### 3.2. Dual Optimization

Even given access to appropriate phages, optimizing a phage cocktail for enhanced breadth of activity along with enhanced depth of activity is not necessarily trivially achieved. For example, a cocktail targeting a single bacterial strain could consist of phages from different cross-resistance groups that possess overlapping host ranges. Such a formulation could provide some depth of activity, as the phages involved do possess overlapping host ranges (and assuming low rates of bacterial mutation to cross resistance); however, it would not necessarily also provide substantial breadth of activity against the multiple strains making up a bacterial species. Alternatively, if two phages possessed completely non-overlapping host ranges, then this cocktail would display no depth of activity against any of the targeted bacterial strains while still demonstrating greater breadth of activity against a given bacterial species, or against more than one species, than would be the case for treatments using either of the constituent phages alone. The case involving more than one species, using three rather than two phages, is illustrated in Figure 1, to the right.

Thus, depending on how a cocktail has been formulated, it may emphasize breadth of activity or depth of activity, but not necessarily both of these simultaneously, even given use of phages inhabiting different cross-resistance groups. Broader spectra of activity and a reduced potential for bacteria to evolve resistance to phages within a cocktail can nevertheless both still be desirable qualities for phage cocktail design [24,56,64]. Improvements, especially of prêt-à-porter formulations toward more than spectrum of activity broadening, should however generally necessitate the inclusion of more phages. Developing such dual activity cocktails, i.e., ones displaying substantial breadth as well as depth of activity—other than by simply randomly adding more phages—may be assisted by also evaluating a cocktail’s breadth of activity explicitly as a function of depth. Strategies for such evaluation are considered in the following sections.

## 4. Developing Dual Spectrum-of-Activity Cocktails

A variety of methods can be used to determine a phage’s host range [7,26,28,65,66,67,68]. These, for example, can be differentiated in terms of (i) ease of use, (ii) potential for false positives, and also (iii) what aspects of phages are being measured, such as bactericidal activity or virion production. We describe here, however, not how phage host ranges are determined but instead, once host-range data exists, how phage cocktails may then be optimized toward enhancing breadth as well as depth of activity. The optimization approaches outlined can be useful especially when working with large host-range data sets. Specifically, they consist of quantitative analyses to determine host-range breadth (using a slight elaboration on how this is typically done), host-range breadth of activity in light of depth of activity (representing an expansion of normal breadth-of-activity determination), and, as considered in the following section, the impact of cross resistance on the latter calculation. Host-range data sets are first converted into a mathematically tractable form and then analyzed using the presented algorithms.

### 4.1. Defining A Phage’s Host Range

No matter what host-range assay one employs, it is crucial—for the quantitative methods described here—to unequivocally distinguish bacteria falling within a phage’s host range (here assigned as ‘1’) from those bacteria falling outside of a phage’s host range (here assigned as ‘0’). More specifically, or operationally, ‘1’ represents a sufficiently negative impact by a specific phage on a specific bacterium. That is, with a score of ‘1’ a phage should be considered to be potentially worthwhile for use against a tested bacterium in phage therapy. For example, a phage may be deemed as being useful toward cocktail development if it is able, within a laboratory setting, to replicate with some level of effectiveness while infecting a potentially targeted bacterial strain.

In terms of experimental outcomes, a ‘1’ could be assigned, for example, to an efficiency of plating found to be above a certain threshold, e.g., 0.1 or 0.5. This binary approach will not necessarily convey the true extent of the complexity of phage–host interactions, however, and for different researchers ‘1’ and ‘0’ may be defined in different ways. Different approaches to defining host-range positives (1s) vs. negatives (0s) might also be compared in terms of the resulting cocktail-development outcomes. In addition, a non-binary scheme might be employed for host-range determinations instead, such as perhaps using actual efficiency of plating values rather than 0s vs. 1s, though this latter approach will not be considered further here. Regardless of the limitations of using a binary approach to describe phage–bacterial interactions, this is what is commonly seen in the phage therapy literature, even if only implicitly. That is, reasonably definite statements are typically made about whether (‘1’) or not (‘0’) a given bacterium should be included in a phage’s host range.

Once phage host-range data has been coded as 1s vs. 0s, for each phage that has been tested against a given panel of bacterial strains, we can then add up the associated 1s and 0s. That phage possessing the highest sum would be designated as having the broadest, or at least most extensive host range against the tested panel of bacterial strains (the distinction between ‘broadest’ and ‘extensive’ being dependent on the diversity characteristics of the bacterial panel used for host-range testing; from here forward, though, we use solely variations on the term ‘broad’). Combining phages with the individually broadest host ranges into a single cocktail will not necessarily result in the broadest cocktail spectrum of activity, however. This is because cocktail breadth of activity is also dependent on the degree to which host ranges do not fully overlap. Taking into account the impact of host-range overlap on phage cocktail breadth of activity is what we consider in the following section, constituting our first approach toward quantitatively analyzing phage cocktail spectrum of activity.

### 4.2. Determining Cocktail Breadth of Activity

To calculate a cocktail’s breadth of activity, one would determine for each tested bacterial strain whether or not the sum of the negative impacts of individual phages making up a given phage cocktail are equal to 0 (e.g., equals 0 + 0 + 0) or instead equal to greater than 0 (e.g., equals 0 + 1 + 0). Thus, the cocktail with the broadest spectrum of activity is that for which [total number of bacterial strains tested] minus [number of bacterial strains ‘hit’ by 0 phages] is greatest. We call the resulting fraction, “Breadth subscript 1”, or “Breadth_1_”, with the subscript “1” indicating that a cocktail’s depth of activity against each bacterium tested must be greater than or equal to 1 to be counted. In other words, this is the fraction of bacteria hit by at least one phage found in a cocktail. This we define as

Breadth_1_ = ([total] − [bacteria not phage impacted])/[total], (1)

where “[total]” is short for [total number of bacterial strains tested]. See Table 2 for a summary of variable and function names, as well as their meanings as used here.

**Figure 2:**
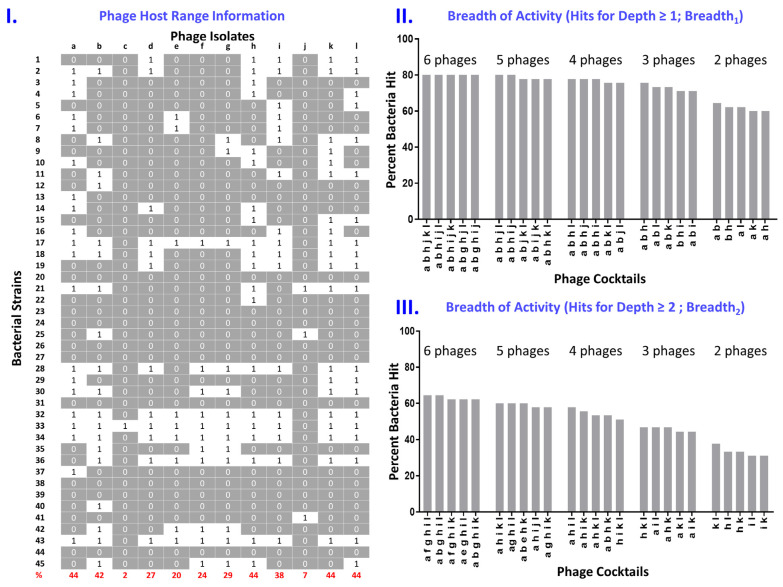
Determination of phage cocktail breadth of activity in light of depth. Panel **I** shows a set of phage host-range data (in columns), with 0s indicating those bacteria (in rows) that are found outside of a phage’s host range and 1s indicating those bacteria that are found within a phage’s host range. Panel **II** shows the breadth of activity of combinations of 6, 5, 4, 3, or 2 phages—as assembled from phage isolates indicated in Panel **I**—that have been identified as cocktails having the broadest spectra (“Percent bacteria hit”). This is in terms of each bacterium being impacted by at least one phage and which thereby represent what we describe as ‘Breadth_1_’. These Breadth_1_ determinations are calculated using Equation (1). Panel **III** is equivalent to Panel **II** except bacteria must be ‘hit’ by at least two phages to be counted, rather than by at least one phage (the latter is calculated instead for Panel **II**). These ‘Breadth_2_’ determinations are calculated using Equation (2).

This ‘Breadth_1_’ approach is how cocktail breadth of activity is generally as well as intuitively determined, though here stated with slightly more formality. Specifically, this is a calculation for the fraction of bacteria tested that are affected by at least one phage making up a given phage cocktail. In Figure 2 we provide a demonstration host-range data set (Panel I) along with breadth of activity (Breadth_1_) for various cocktails consisting of different phage combinations (Panel II).

#### Multiple Usages of Numbering, a Potential Source of Confusion

Note that we are using numbers and numbering here in multiple ways. For example, and most egregiously, for the number ‘1’ these include (a) as equivalent to ‘true’ in a binary relationship (i.e., 1 vs. 0); (b) as a number of cross-resistance groups populated by the phages found in a cocktail; (c) as the name of a bacterial strain such as in Figure 2; (d) potentially as a subscript also referring to the name of a bacterial strain (see Depth*_n_*, Table 2); (e) as a description of a depth of activity of a phage cocktail for a specific bacterial strain (e.g., “Depth = 1”, Figure 1); (f) as a subscript (Breadth_1_ and True_1_) referring to the minimum depth of activity counted also for a cocktail (Table 2); and (g) as the names of the first Equation, the first figure, and the first table. In addition, in terms of binary relationships, a ‘1’ could refer not only to a bacterium being found within a phage’s host range but also whether a given bacterial strain is hit by some minimum number of phages, i.e., as returned by True_1_ and True_2_, etc. (Table 2). Please, therefore, take care when interpreting numbers and numbering usage.

### 4.3. Taking into Account Cocktail Depth of Activity

In calculating a cocktail’s breadth of activity, each bacterium needs to be hit by at least one phage. Indeed, this idea is built into the above-described algorithm (Equation (1)) in distinguishing bacterial strains that are affected by zero phages (Depth = 0) from those that are affected by *more than zero* phages (Depth ≥ 1, defining Breadth_1_). Toward greater cocktail depth of activity, however, each targeted bacterial strain instead must be hit by *more than one* phage (Depth ≥ 2, defining instead a Breadth_2_). For personalized phage treatment, i.e., sur-mesure, such a determination can be somewhat trivial. This is because one need identify only, e.g., two phages that target a single, specific bacterial pathogen.

Spectrum of activity determinations taking depth of activity into account can become more complex toward development of prêt-à-porter phage cocktails relative to development of cocktails for sur-mesure use, that is, when targeting more than one bacterial strain of a single bacterial species rather than only a single bacterial strain. Consistent with the method outlined in the previous section on calculating phage cocktail breadth of activity (Equation (1)), the approach nonetheless can still be conceptually simple. Specifically, in terms of numbers rather than fractions of bacterial strains, it becomes [total number of bacterial strains tested] minus [number of bacterial strains hit by fewer than two phages]. This constitutes the basis of our second presented means of quantitatively analyzing cocktail spectrum of activity, which we call “Breadth_2_”:

Breadth_2_ = ([total] − [bacteria impacted by less than 2 phages])/[total], (2)

where “impacted” is short for “negatively impacted” and “[total]” again is short for [total number of bacterial strains tested] (with the “2” in Breadth_2_ referring to depths of activity of ≥2).

For even greater depth of activity, “less than 2” in Equation (2) (defining Breadth_2_) can be replaced with “less than 3” (defining a Breadth_3_), etc. The fraction of bacteria in our hypothetical panel impacted by at least two phages, for different phage combinations making up cocktails, is presented in Panel III of Figure 2 vs. as impacted by at least 1 phage (Panel II). In order to reduce the effort required to do breadth and depth calculations using Equation (2) as well as Equation (1), especially when working with large host-range data sets, we have developed an online, JavaScript-based, “Phage cocktail optimizer” calculator [31].

### 4.4. Additional Observations

Note, in Figure 2, the generally lower percentage of bacteria hit with a Depth of ≥2 (Panel III) vs. a Depth of ≥1 (Panel II), i.e., Breadth_2_ vs. Breadth_1_. This combination of greater breadth given lesser depth should represent a trend, and this is because by increasing depth we are imposing an additional constraint on a cocktail’s functionality. Indeed, as a general rule the breadth of activity of a cocktail possessing a greater depth of activity should never be greater than the breadth of activity of a cocktail possessing a lesser depth of activity. For example, breadth at a Depth such as of ≥1 will always include, e.g., those bacteria for which Depth instead is ≥2, but not vice versa. Based on observation of randomly generated host-range data sets (not presented), these suppositions appear to be born out. In short, and not unexpectedly, it should be harder to hit the same number of bacteria with two different phage types than with just a single phage type.

Less foreseen in Figure 2 is the observation that the phage combinations that make up the optimal phage cocktails in Panel III (Depth ≥ 2) are not identical to the optimal phage cocktails in Panel II (Depth ≥ 1). This result was also observed in the simulated but not presented host-range sets mentioned above and likely is a consequence of the general assertion that it is difficult to simultaneously optimize both a cocktail’s breadth of activity and a cocktail’s depth of activity. Within a heterogeneous mixture, that is, it is likely that some phage combinations are better at supplying breadth of activity than they are at supplying depth of activity, while other phage combinations may be better at supplying depth of activity. A given phage cocktail optimized for breadth of activity, in other words, will not necessarily also be optimal for substantial depth of activity across all bacterial strains targeted, or vice versa, merely by chance [53].

## 5. Taking Cross Resistance into Account

**Figure 3:**
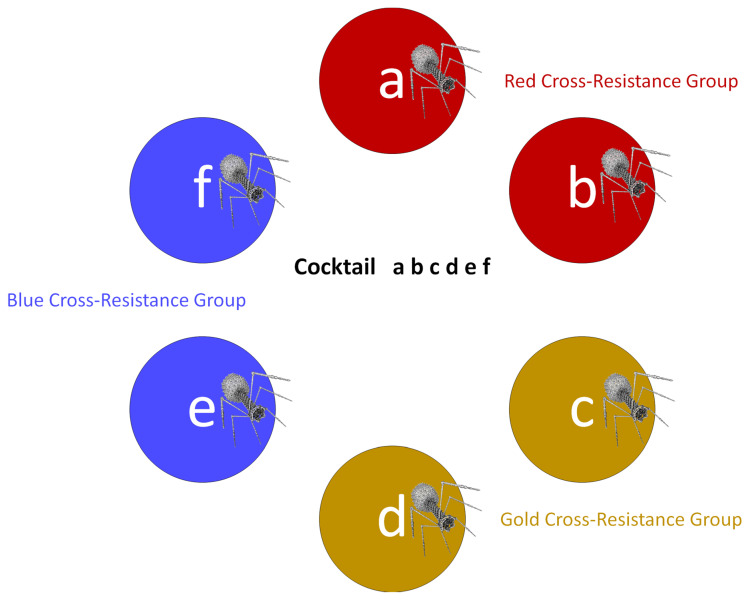
Combining phages found in both different and the same cross-resistance groups into a single phage cocktail. Phages a and b, c and d, and e and f are found within three different cross-resistance groups, red, gold, and blue, respectively. The presented cocktail, dubbed “a b c d e f”, consists of six phages and three cross-resistance groups, with constituent phages thus found overall in combinations of the same and different cross-resistance groups.

Phage cocktail depth of activity is a function not only of raw numbers of phages impacting a bacterial strain but also of the potential for that bacterium to mutate to cross resistance to the different phages used. Indeed, only phage combinations that have all been drawn from different cross-resistance groups should be used toward Depth ≥ 2 cocktail breadth-of-activity calculations (Breadth_2_; Equation (2)). Limiting cocktails only to phages found in different cross-resistance groups, however, may be too constraining for cocktail development toward enhancement solely of breadth of activity (Breadth_1_). This section therefore elaborates on Equation 2-type calculations to assess the breadth of activity of individual cocktails that consist of mixtures of phages which are found in various combinations of both different and the same cross-resistance groups (Figure 3).

### 5.1. Breadth_2_ Calculations for All Phage Combinations

To develop phage cocktails optimized for both breadth and depth of activity—while also taking cross resistance into account, but with emphasis not solely on avoiding cross resistance—it is necessary to expand upon Equation 2-type analyses. This involves including in calculations all phages found in a possible cocktail, even if not all phages fall within different cross-resistance groups. The process consists of determining the depth of activity associated with every combination of phages that are found in different cross-resistance groups for every bacterial strain tested. For instance, phages a, c, and f from Figure 3 would represent one such combination, each of which is found in a different cross-resistance group, while phages b, d, and f would constitute another such combination.

The calculations for each of these individual phage combinations and bacterial strains tested—which can be quite extensive in total number—we describe as giving rise to individual Depth*_n_* values. Here, the subscript ‘*n*’ is the name of a single, tested bacterial strain, e.g., ‘14′ from Figure 2, panel I. The total number of Depth*_n_* calculations in turn would be equal to the number of phage combinations multiplied by the number of bacterial strains tested. For Figure 3, there would be a total of eight combinations of phages, i.e., ‘sub-cocktails’, consisting of a c e, a d e, a c f, a d f, b c e, b c f, b d e, and b d f. From Figure 2, panel I, there would a total of 45 strains. Thus, the total number of individual Depth*_n_* calculations in the case of Figure 3 could be 45 × 8 = 360, e.g., Depth_11_ (a c e), Depth_17_ (b d f), etc.

**Figure 4:**
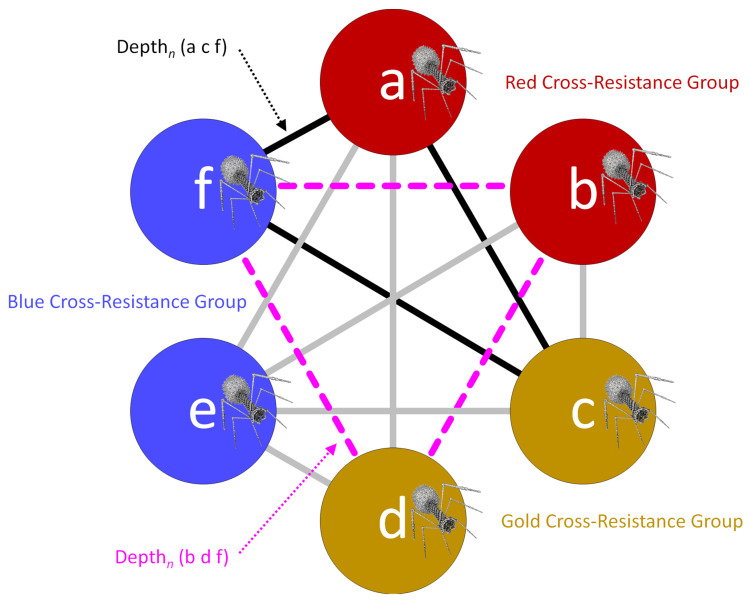
Calculating Breadth_2_ without all phages being found in different cross-resistance groups. Phages a through f are found in three different cross-resistance groups, red, gold, and blue, i.e., as presented equivalently in Figure 3. Thick lines between phages serve as guides as to what phage combinations (sub-cocktails) would be involved in Equation 2-based calculations. As examples, note the lines between phages a, c, and f (black-solid) or between phages b, d, and f (purple-dashed). The respective Depth_n_ (a c f) and Depth_n_ (b d f) values are either a 0, 1, 2, or 3. This is 0 if no phages from a given combination hit an individual tested bacterial strain (n), 1 if only one hits, 2 if two hit, and 3 if three hit. Considering all six of these phages, then Breadth_2_ (a b c d e f) = { $\sum_{1}^{n}$ True_2_ [Depth*_n_* (a c e), Depth*_n_* (a c f), Depth*_n_* (a d e), Depth*_n_* (a d f), Depth*_n_* (b c e), Depth*_n_* (b c f), Depth*_n_* (b d e), Depth*_n_* (b d f)] }/*n*. This is every possible combination of phages acting on every bacterial strain tested, where each phage making up an individual phage combination has been sourced from a different cross-resistance group. The function True_2_ returns a value of 1 if any Depth*_n_* value for a tested bacterial strain is a 2 or higher. Alternatively, True_2_ returns a value of 0 for a given bacterial strain if that strain is not hit by at least two phages sourced from different cross-resistance groups. Breadth_2_ (a b c d e f) thus will have a value of between 0 and 1, ranging from no bacteria hit by at least two different phages sourced from different cross-resistance groups to all bacterial strains tested hit by at least two different phages sourced from different cross-resistance groups. For example, Breadth_2_ (a b c d e f) = 0.5 would mean that half of the bacteria tested were individually hit by at least two different phages sourced from two different cross-resistance groups, while the other half were not. This specific example is not solvable as presented, however, as no actual phage host-range data is provided. See instead the main text, Figure 5, and then Figure 6 and Table S1.

For the three phages a, c, and f, if the host range of only phages a and c but not phage f includes a given tested bacterium, call the latter ‘14′, then we would describe a Depth_14_ for those three different phages together as being equal to 2, as only two of those three phages hit the tested bacterium. Thus, in this example, Depth_14_ (a c f) = 2. The next step involves determining whether at least one of the possible combinations of phages sourced from different cross-resistance groups results in a Depth*_n_* of *at least* 2 also for each bacterial strain tested, e.g., such as is the case for Depth_14_ (a c f). We describe this latter step as representing a ‘True_2_’ function, which returns a binary value of 1 if at least one Depth*_n_* value for a given tested bacterial strain is equal to at least 2 (where the latter is the meaning of the ‘2’ subscript in True_2_). The number of True_2_ positive results (that is, True_2_ = 1) across all bacterial strains tested, divided by the total number of bacterial strains tested (*n*), defines this more elaborately determined Breadth_2_ value, which in this case we call Breadth_2_ (a b c d e f). We illustrate the first steps in how to do this in Figure 4, again using the hypothetical six-phage cocktail introduced in Figure 3.

### 5.2. Example of Breadth_2_ Calculations Based on Limited Host-Range Data

Including host range data in calculations requires additional bookkeeping, so we introduce this with a simpler example than that presented in Figure 3 and Figure 4. Consider, therefore, a phage cocktail consisting of three phages, x, y*, and z, where phages x and z are found in the same cross-resistance group while phage y* is found in a different cross-resistance group (with the asterisk associated with the ‘y’ phage name used here to emphasize this cross-resistance group distinction). Equivalent to Figure 4, the depth-of-activity calculation is accomplished on a per-tested-bacterium basis for individual combinations of phages (sub-cocktails) for which cross-resistance does not readily occur, i.e., the sub-cocktail consisting of phages x and y* along with the sub-cocktail consisting of phages y* and z. Thus,

(3) $${\mathrm{Breadth}}_{2}\;(x\;y*\;z)=\text{\{}\sum_{1}^{n}\;{\mathrm{True}}_{2}\;[{\mathrm{Depth}}_{n}\;(x\;y*),\;{\mathrm{Depth}}_{n}\;(y*\;z)]\;)/n,$$

where 1 through *n* are individual bacterial strains tested of a total number, *n*; Depth*_n_* (x y*) is set to 2 if both phages x and y* hit bacterial strain *n*; Depth*_n_* (y* z) is also set to 2 if both phages y* and z hit bacterial strain *n*; and True_2_ [Depth*_n_* (x y*), Depth*_n_* (y* z)] returns a value of 1 for a given bacterial strain, *n*, if either Depth*_n_* (x y*) or Depth*_n_* (y* z)—and thereby activity depth for the combination of phages x, y*, and z for that bacterial strain—is equal to at least 2 (which, as noted, is the meaning of the ‘2′ subscript in both True_2_ and Breadth_2_). This maximum calculated depth of activity for an individual bacterial strain—as equal in this example to Depth*_n_* (x y*) or Depth*_n_* (y* z), or both—we describe, as noted, as a cocktail’s ‘activity depth’ for that strain.

**Figure 5:**
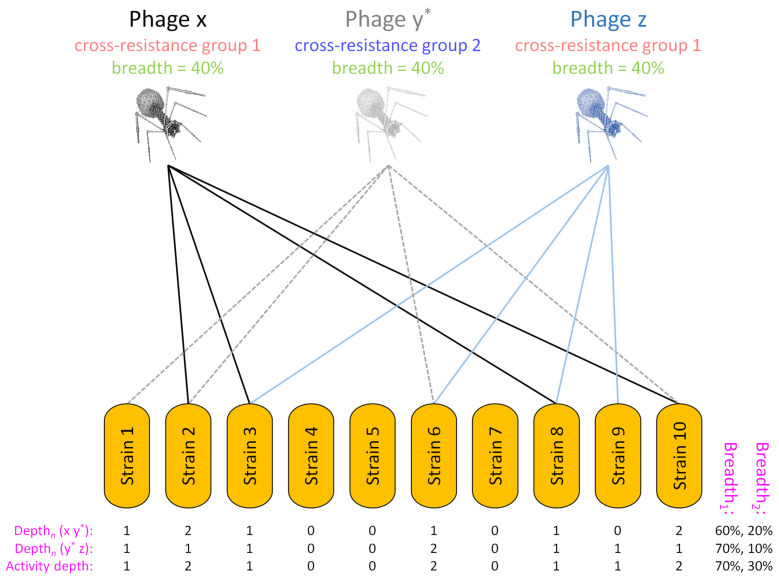
Determination of Breadth_2_ for phages found in both the same and different cross-resistance groups. Different bacterial strains, ‘1’ through ‘10′, are indicated in orange toward the bottom of the figure. Lines indicate that a bacterial strain is found within the host range of either phages x, y*, or z, with phage y* as indicated found in a different cross-resistance group from phages x and z. Depths of activity for each bacterial strain are indicated as the numbers 0, 1, or 2. These values cannot be greater than 2 in this example because there are only two phage cross-resistance groups, phages x and z versus phage y*. For the sub-cocktail consisting of phages x and y* (top row) these are Depth*_n_* (x y*) values. For the sub-cocktail consisting of phages y* and z (middle row) these are Depth*_n_* (y* z) values. Finally, for the combination of phages x, y*, and z (the full cocktail; bottom row) these are the activity depths for each individual bacterial strain tested. Shown to the right are percentages of bacterial strains hit for the different phage combinations. This is by at least one phage (bigger percentage = Breadth_1_, e.g., 60% for the top row) or instead by at least two phages from different cross-resistance groups (smaller percentage = Breadth_2_, e.g., 20% also for the top row). Carrying out calculations for each bacterial strain tested, the bottom digit in a column (“Activity depth”) is a 1 if at least one previous digit in the same column is a 1 and also no previous digits are larger than a 1 (this criterion is met in the figure for bacterial strains 1, 3, 8, and 9). Alternatively, the bottom digit is a 2 if at least one previous digit is a 2 (or in the case of different examples from this one, potentially instead a number greater than a 2). A Depth of 2 thus is the case for bacterial strains 2, 6, and 10. To calculate Breadth_1_ (x y* z), simply count the fraction of 1s or higher found across the bottom row, i.e., as per the True_1_ function, which is a process that is equivalent to solving Equation (1) as well as Equation (4). This comes out to 7 out of 10 or 70%. To calculate Breadth_2_ (x y*) or Breadth_2_ (y* z), both of which are based on Equation (2), count only the fraction of 2s as well as any higher values if they were present. Thus, e.g., Breadth_2_ (x y*) = 2/10 or 20%. To calculate Breadth_2_ (x y* z), as based on Equation (3), do the same, i.e., count the fraction of 2s or higher as per the True_2_ function, but for the bottom row only. Thus, Breadth_2_ (x y* z) = 3/10 or 30%. In other words, in this example a total of three bacterial strains of ten are hit by two phages from different cross-resistance groups. This is the breadth of activity of this cocktail in light of an activity depth for three specific bacteria that is sufficient to reduce their potential to mutate to phage resistance, of a total of ten bacterial strains tested.

Contrasting activity depth, Breadth_2_ (x y* z) is calculated, whether by Equation (2) or using Equation (3), across *all* bacterial strains tested. A given cocktail thus has a specific, determinable activity depth for each individually-tested bacterial strain, which is a function of the number of different cross-resistance groups the phages hitting a given bacterium are a part of, while Breadth_2_ is the fraction of bacteria tested for which a cocktail has an activity depth of at least 2. In Figure 5, we provide an example of how activity depth is calculated for the cocktail consisting of phages x, y*, and z (resulting in the bottom row) and how Equation (3), that is, Breadth_2_ (x y* z), may be solved (resulting in the far-right value, also in the bottom row).

In the legend of Figure 5, Breadth_1_ (x y* z) is also calculated, as 7/10 or 70% (second to last column, bottom row). This is solved as,

(4) $${\mathrm{Breadth}}_{1}\;(x\;y*\;z)=\text{\{}\sum_{1}^{n}\;{\mathrm{True}}_{1}\;[{\mathrm{Depth}}_{n}\;(x\;y*),\;{\mathrm{Depth}}_{n}\;(y*\;z)]\;)/n,$$

where True_1_ [Depth*_n_* (x y), Depth*_n_* (y z)] returns a 1 if either Depth*_n_* (x y*) or Depth*_n_* (y z) is equal to 1 or greater. Therefore, {$\sum_{1}^{n}$ True_1_ [Depth*_n_* (x y*), Depth*_n_* (y* z)]} is equal to {[total] − [bacteria not phage impacted]} as found in Equation (1). Breadth_2_ (x y* z), by contrast, is calculated in Figure 5 and found to be equal to only 3/10, or 30% (last column, bottom row). This disparity between Breadth_1_ (x y* z) (= 70%) and Breadth_2_ (x y* z) (= 30%) is qualitatively similar to how calculated Breadth_2_ values (panel III) are somewhat smaller than calculated Breadth_1_ values (panel II), both as presented in Figure 2.

### 5.3. Example of Breadth_2_ Calculations Based on More Extensive Cross-Resistance Data

**Figure 6:**
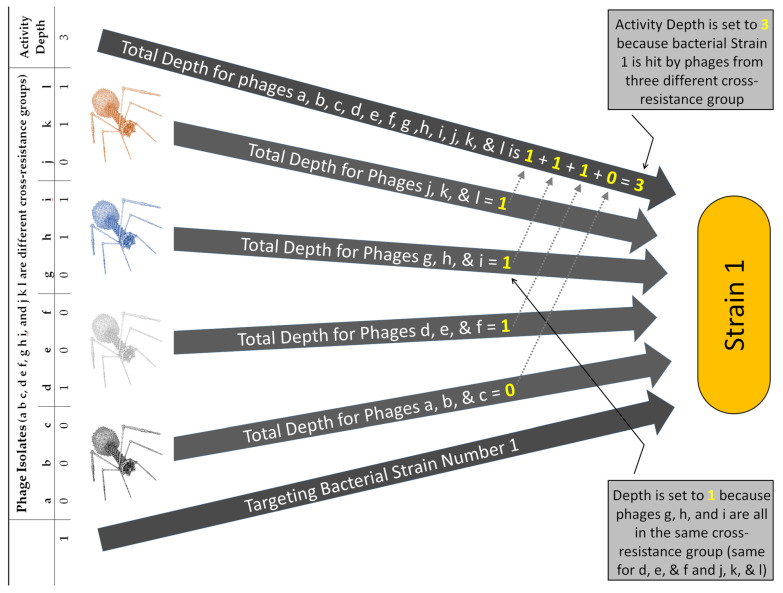
Example of calculation of activity depth taking into account multiple cross-resistance groups with diverse phage membership. Shown to the left are the first rows of Table S1, Supplementary Materials. The number “1” found in the lower, left-hand corner refers to bacterial strain number 1, as indicated within the lower arrow. Demonstrated also within the arrows (top) is how an activity breadth of 3 is arrived at for bacterial strain number 1. Note that the “Total depth” for each cross-resistance group cannot exceed a value of 1 and thus is equal to either 0 or 1.

In the previous two Sections (5.1 and 5.2) we demonstrated how cocktail breadth of activity can be determined in light of depth of activity across multiple bacterial strains, with cocktail phages diverse in terms of their membership in both different and the same cross-resistance groups. To ease determinations based on more extensive data sets, we have developed an online, JavaScript-based app for performing these Equation 3-type Breadth_2_ calculations [32]. To further illustrate both such calculations and their results, we have taken the host-range data presented in Panel I of Figure 2, arbitrarily divided it into four cross-resistance groups, and then applied this app to the data. The resulting analysis is presented in Table S1 (Supplementary Materials) and also illustrated in part in Figure 6.

It is also possible to calculate Breadth_2_ for that cocktail visually by using Table S1, which in part is summarized in Figure 6. This is because the analysis consists simply of determining whether at least one phage that is found within each cross-resistance group hits the targeted bacterial strain. One then counts the number of cross-resistance groups that contain at least one such phage and the resulting sum is equal to the activity depth for a given bacterial strain. To obtain Breadth_2_, one then divides [number of bacterial strains with activity depth ≥ 2] by [all bacterial strains tested]. This, as a reminder, is the fraction of bacterial strains hit by least two phages sourced from different cross-resistance groups, and therefore for which the potential for evolution of phage resistance is reduced relative to the total number of bacterial strains targeted. The calculation is also essentially a restatement of Equation (2) while taking into account cross resistance, and gives a result that is equivalent to that provided by Equation (3). Similar calculations can be made from this data set (Table S1) to obtain a Breadth_3_ or Breadth_4_. Further discussion of these calculations can be found in Section S1 (Supplementary Materials).

As a reminder, these are arbitrary cross-resistance data sets that are being analyzed in both Table S1 and Figure 5 and Figure 6. Therefore, no inferences can be made from the presented percentages other than that it is expected that Breadth_1_ will tend to be larger than Breadth_2_ and that Breadth_2_ will tend to be larger than Breadth_3_, etc. The general approach to calculating phage cocktail breadth of activity in light of depth of activity, taking cross resistance into account, will however remain the same when analyzing real host-range and cross-resistance data.

### 5.4. Complications Involving Cross Resistance

Unfortunately, just what combinations of phages might constitute a cross-resistance group is not always straightforward. For example, a phage that is able to target two different bacterial receptor molecules—such as phage T2 infecting *E. coli* B, as mentioned above [59], and see also [60] as well as [46,61,62]—may not be associated with any phage cross-resistance groups so long as targeted bacteria possess both receptors. That is, mutation to resistance to a phage that uses a single phage receptor may be unlikely to result also in resistance to a phage that can adsorb using either of two bacterial surface molecules as receptors. With bacterial hosts that possess only one of those receptors, however, the same phage should be readily found within one or more cross-resistance groups.

In addition, there is the potential for different phages to use the same receptor molecules for adsorption, but not the same parts of these receptor molecules, e.g., as can be the case when bacterial LPS serves as a phage receptor [69]. In this case, some mutations to resistance to one phage may not result in resistance to other phages that use the same molecule as a receptor, even if both phages otherwise are found in the same cross-resistance group. Thus, sometimes cross resistance can be *asymmetrical*, with mutations to resistance to one phage resulting in resistance to a second phage (thus displaying cross resistance), but with not all mutations to resistance to the second phage resulting in resistance to the first phage [27]. Mutations to phage resistance that do not involve bacterial surface molecules likely also occur, the existence of which is suggested by the results of Kortright et al. [70].

Together these various phenomena can confound determinations of cross-resistance groups. The above calculations incorporating issues of cross resistance into cocktail dual optimization therefore will not necessarily always be the last word on the enhancement of prêt-à-porter cocktail depth of activity.

## 6. Discussion

Phage cocktails may be developed toward more general, prêt-à-porter use. Emphasis in this case is on enhancing spectrum of activity breadth, thereby allowing for more effective empirical use. Among these off-the-shelf products are those that could be distributed commercially and which therefore must target multiple bacterial strains, ones belonging to either a single or instead multiple bacterial species. Phage cocktails alternatively may be developed toward personalized, sur-mesure use, targeting especially single bacterial strains, with a possible goal of enhancing cocktail activity depth rather than breadth. That is, they would be using a cocktail rather than a monophage in order to better combat the potential for evolution of phage resistance by a targeted bacteria strain.

As we have emphasized, these two goals of cocktail breadth of activity and cocktail depth of activity are not identical. Of particular interest would be combining both properties into individual, off-the-shelf products, particularly ones targeting multiple strains making up a single bacterial species. These bacterial species could include, for example, *Acinetobacter baumannii* [71,72], *Escherichia coli* [73,74], *Klebsiella pneumoniae* [75], *Mycobacterium abscessus* [76,77], *Pseudomonas aeruginosa* [78,79,80,81,82,83,84], or *Staphylococcus aureus* [3,85,86,87,88,89,90,91,92,93,94], all of which, as cited, have been treated clinically using phage cocktails.

Toward the goal of developing especially prêt-à-porter cocktails that are enhanced in both their breadth and depth of activity, we have presented an in silico approach to cocktail characterization that emphasizes not just increasing the diversity of bacterial strains targeted but increasing the number of bacterial strains targeted by phages that have been drawn from different cross-resistance groups. This is rather than leaving solely to chance the attainment, by prêt-à-porter cocktails, of both significant breadth and significant depth of activity.

## 7. Conclusions

What is perhaps unique about the use of phage cocktails as a form of combination therapy is their potential to be designed without explicit consideration of combating bacterial resistance evolution. That is, as highlighted here, phage cocktails can be designed with an emphasis on enhancing spectrum of activity breadth with little active consideration toward also enhancing spectrum of activity depth. With purely chemotherapeutic combination therapies, by contrast, the primary emphasis—in the treatment especially of specific, identified pathogens such as *M. tuberculosis*—instead is generally on enhancing what here we have described as spectrum of activity depth. Of course, the reason for this disparity is the often narrow spectrum of activity breadth displayed by individual phages, in combination with intentions to develop phage preparations that can be used commercially, empirically, and/or off the shelf. Thus, to formulate phage cocktails to also combat the evolution of resistance, additional attention is required, as we have considered here. We have shown especially that cocktail depth of activity can be used as a criterion for calculating a cocktail’s breadth of activity, including in light of bacterial cross resistance to different phages. We note in particular, however, that increasing cocktail breadth in light of cocktail depth appears to be more challenging than expanding cocktail breadth alone.

## Figures and Tables

**Table 1:** Contrasting cocktails based on spectrum of activity breadth and depth.

Cocktail Properties:	Single Strain	Single Species	Multiple Species
Targeting single patient?	Yes	No ^1^	No ^1^
Breadth of activity goal?	Low	Medium	High
Depth of activity goal?	High (ideally)	Not necessarily high	Not necessarily high ^2^
Prêt-à-porter ^3^ as goal?	Not necessarily	Yes	Yes (potentially ^1^)
Sur-mesure ^4^ as goal?	Yes	No ^1^	No ^1^
Personalized medicine?	Yes	No ^1^	No ^1^
Minimizing Resistance?	Readily achievable	Partially achievable ^5^	Difficult with breadth
Cross resistance an issue?	Yes (if depth is goal)	Yes (if depth is goal)	No (for between species)

^1^: Unless for treatment of mixed infections consisting of multiple strains of the same species or multiple species; ^2^: “High” meaning > 1, i.e., two or more phages together impacting a substantial fraction of bacteria targeted; ^3^: Idiomatically meaning ‘off the shelf’, i.e., the typical goal for phage cocktails for commercial use; ^4^: Meaning ‘custom made’, e.g., a formulation for personalized medicine cocktail use; ^5^: Such as a large fraction of bacterial strains being targeted by more than one phage making up a cocktail.

**Table 2:** Summary of terms and notations used.

Notation	Usage	Units	Meaning
“Hit”	Term	NA	Referring to a bacterium being found within a phage’s host range
Sub-cocktail	Term	NA	Referring to a subset of the phage types making up a cocktail, especially where none of that subset of phage types are found in the same cross-resistance groups; sub-cocktails are used in Depth*_n_* calculations toward activity depth determinations
0	Number	NA	Refers to a binary output meaning ‘false’ as well as a description of depth of activity, in the latter case meaning that a bacterium is not hit by a given phage or is hit by no phages found in a cocktail
1	Number	NA	Refers to binary outputs meaning ‘true’, as well as a description of depth of activity, in the latter case meaning that a bacterium is hit either by only a single phage found in a cocktail or by at least one phage; we use ‘1’ also as a stand-in for the name of a bacterial strain
Letters, lower case	Abbreviations	NA	Phage names, e.g., a, b, c, … j, k, l as designating columns in Figure 2, panel I
Numbers	Abbreviations	NA	Bacterial strain names, e.g., 1, 2, 3, … *n* as designating rows in Figure 2, panel I
Breadth_1_	Variable	Fraction of bacteria tested	Breadth of activity of a phage cocktail without taking depth of activity into account; fraction of bacteria hit by at least one phage in a phage cocktail; see Equations (1) and (4)
Breadth_2_	Variable	Fraction of bacteria tested	Breadth of activity of a phage cocktail taking depth of activity into account; fraction of bacteria hit by at least two phages found in different cross-resistance groups and thus more limited in their potential to evolve phage resistance; see Equations (2) and (3)
Breadth_3_	Variable	Fraction of bacteria tested	Same as Breadth_2_ except fraction of bacteria hit by three or more phages; Breadth_4_ would be by four or more phages; the general case we indicate as Breadth*_x_*
*n*	Variable	Bacteria	A given tested bacterial strain or total number of tested bacterial strains
Activity depth	Variable	Number of phages of a cocktail	Number of phage cross-resistance groups that hit a given, tested bacterial strain, e.g., equal to 2 if hit by phages from two different groups as based on Depth*_n_* calculations made for all sub-cocktails; see Figures 5 and 6 and Table S1
Depth*_n_*	Variable	Number of phages in a sub-cocktail	Depth of activity impacting a given, tested bacterial strain, *n*, that is associated with a specific sub-cocktail of phage types, e.g., phages a, c, and f, and thus Depth*_n_* (a c f); can be used in Breadth*_x_* determinations; see Equations (3) and (4) and Figures 4 and 5
True_1_	Function	Binary (0 or 1)	Returns a value of 1 for a given tested bacterial strain if at least one phage in a cocktail hits that bacterial strain; can be used in Breadth_1_ determinations; see Equation (4) and Figure 5
True_2_	Function	Binary (0 or 1)	Returns a value of 1 for a given tested bacterial strain if at least two phages in a cocktail from different cross-resistance groups hit that bacterial strain; can be used in Breadth_2_ determinations, i.e., see Equation (3) and Figures 4 and 5; True_3_ is for at least three phages, etc.
x y z	Notation	NA	Description of the phages making up a cocktail, in this case phages x, y, and z; no indication of phage cross-resistance grouping is explicitly provided by this notation; commas between letters are implied but are not shown in order to reduce clutter

## Data Availability

Not applicable.

## Supplementary Materials

The following are available online at https://www.mdpi.com/article/10.3390/ph14101019/s1. S1: Example of Breadth_2_ calculations based on more extensive cross-resistance data; Table S1: Depth of activity against various bacterial strains, taking cross resistance into account; S2: www.phage-therapy.org/calculators/cocktail_optimizer.html; S3: www.phage-therapy.org/ scripts/cocktail_optimizer.js; S4: www.phage-therapy.org/calculators/xresistance_avoider.html; S5: www.phage-therapy.org/scripts/xresistance_avoider.js.
