# Supplementary material for: Phage Cocktail Development for Bacteriophage Therapy: Toward Improving Spectrum of Activity Breadth and Depth
Source: Pharmaceuticals (Basel). 2021 Oct 3;14(10):1019. doi: 10.3390/ph14101019 (PMC8541335; doi:10.3390/ph14101019)
Supplement: Supplementary file 1 [file pharmaceuticals-14-01019-s001.zip › pharmaceuticals-1395630-supplementary.pdf]

pharmaceuticals

MDPI

Opinion

Phage Cocktail Development for Bacteriophage Therapy: Toward Improving Spectrum of Activity Breadth and Depth, Supplementary Materials

Stephen T. Abedon[1,*,†], Katarzyna M. Danis-Wlodarczyk[2,†] and Daniel J. Wozniak[1,2]

[1] Department of Microbiology, The Ohio State University, Columbus, OH 43210, United States; daniel.wozniak@osumc.edu
[2] Department of Microbial Infection and Immunity, The Ohio State University, Columbus, OH 43210, United States; danis-wlodarczyk.1@osu.edu
† Co-first authors
* Correspondence: abedon.1@osu.edu

Citation: Abedon, S.T.; Danis-Wlodarczyk, K.M.; Wozniak, D.J. Phage Cocktail Development for Bacteriophage Therapy: Toward Improving Spectrum of Activity Breadth and Depth. *Pharmaceuticals* **2021**, *14*, 1019. https://doi.org/10.3390/ph14101019

Academic Editor: Gill Diamond

Received: 09 September 2021
Accepted: 27 September 2021
Published: 14 October 2021

Publisher's Note: MDPI stays neutral with regard to jurisdictional claims in published maps and institutional affiliations.

Copyright: © 2021 by the authors. Licensee MDPI, Basel, Switzerland. This article is an open access article distributed under the terms and conditions of the Creative Commons Attribution (CC BY) license (https://creativecommons.org/licenses/by/4.0/).

Abstract: Phage therapy is the use of bacterial viruses as antibacterial agents. A primary consideration for commercial development of phages for phage therapy is the number of different bacterial strains that are successfully targeted, as this defines the breadth of a phage cocktail's spectrum of activity. Alternatively, phage cocktails may be used to reduce the potential for bacteria to evolve phage resistance. This, as we consider here, is in part a function of a cocktail's 'depth' of activity. Improved cocktail depth is achieved through inclusion of at least two phages able to infect a single bacterial strain, especially two phages against which bacterial mutation to cross resistance is relatively rare. Here, we consider the breadth of activity of phage cocktails while taking both depth of activity and bacterial mutation to cross resistance into account. This is done by building on familiar algorithms normally used for determination solely of phage cocktail breadth of activity. We show in particular how phage cocktails for phage therapy may be rationally designed toward enhancing the number of bacteria impacted while also reducing the potential for a subset of those bacteria to evolve phage resistance, all as based on previously determined phage properties.

Table of Contents (internal links) to Supplemental Materials

- S1. Example of $Breadth_2$ calculations based on more extensive cross-resistance data
- Table S1
- S2. www.phage-therapy.org/calculators/cocktail_optimizer.html
- S3. www.phage-therapy.org/scripts/cocktail_optimizer.js
- S4. www.phage-therapy.org/calculators/xresistance_avoider.html
- S5. www.phage-therapy.org/scripts/xresistance_avoider.js

Coding also can be accessed by copying and pasting the indicated links.

S1. Example of Breadth$_2$ calculations based on more extensive cross-resistance data

We have taken the host-range data presented in Panel I of Figure 2, arbitrarily divided it into four cross-resistance groups, and then placed that data in Table S1 to illustrate calculations of host-range depth in light of cross resistance using a more complex data set.

Note that the analysis may be done based solely on visual inspection of the host-range data that is presented in Table S1. This is achieved by reading across rows, as described in the footnote to that table. See Figure 6 of the main article for further explanation. In addition, note that equivalent data is presented slightly differently in Table S1 vs. Figure 5. Specifically, the right-most column of digits in Table S1 ("Activity depth") is equivalent to the lower-most row in Figure 5 (also "Activity depth").

In Table S1, the relative breadth of activity for a depth of 1 or more is 80% (36/45) = Breadth$_1$. This is as calculated across all of the phages tested. That is, in the last column, which indicates depth of activity as calculated for each bacterial strain tested, i.e., "Activity depth", there are a total of 9 zeroes, so 36 of 45 activity depths are greater than or equal to 1. This means in turn that 4 out of 5 bacteria found in the test panel are susceptible to at least one phage found in this hypothetical 12-phage cocktail.

For a depth of 2 or more, the breadth is 67% (30/45) = Breadth$_2$ across all of the phages tested, which is determined also simply by counting up the numbers of values, in this case values of 2 or higher, that are found in the right-most column (30), and then dividing by the total number of values (45). This means that evolution of phage resistance would be expected to be interfered with for two-thirds of the bacteria tested, as due to these bacteria each being hit by two or more phages sourced from different cross-resistance groups.

For depths of 3 or more, or instead of 4, the breadth of activity is 53% (24/45) (= Breadth$_3$) and 24% (11/45) (= Breadth$_4$), respectively. This means for these fractions that the potential for bacteria to evolve phage resistance, as hit by a minimum of 3 or 4 phages sourced from different cross-resistance groups rather than a minimum of 2, should be even lower given exposure to these 12 phages. Activity depths of greater than 4 in this example are not possible since only 4 phage cross-resistance groups are being considered.

Table S1: Depth of activity against various bacterial strains, taking cross resistance into account.

	**Phage Isolates** (a b c, d e f, g h i, and j k l are different cross-resistance groups)												**Activity Depth**[1]
**Bacterial Strains**	**a**	**b**	**c**	**d**	**e**	**f**	**g**	**h**	**i**	**j**	**k**	**l**	
**1**	0	0	0	1	0	0	0	1	1	0	1	1	3
**2**	1	1	0	1	0	0	0	1	1	0	1	1	4
**3**	1	0	0	0	0	0	0	1	0	0	0	0	2
**4**	1	0	0	0	0	0	0	1	0	0	0	1	3
**5**	0	0	0	0	0	0	0	0	1	0	0	1	2
**6**	1	0	0	0	1	0	0	0	1	0	0	0	3
**7**	1	0	0	0	1	0	0	0	1	0	0	0	3
**8**	0	1	0	0	0	0	1	0	1	0	1	1	3
**9**	0	0	0	0	0	0	1	1	0	0	1	0	2
**10**	1	0	0	0	0	0	0	1	0	0	1	0	3
**11**	0	1	0	0	0	0	0	0	1	0	1	1	3
**12**	0	1	0	0	0	0	0	0	0	0	0	0	1
**13**	1	0	0	0	0	0	0	0	0	0	0	0	1
**14**	1	0	0	1	0	0	0	1	0	0	0	0	3
**15**	0	0	0	0	0	0	0	1	0	0	1	1	2
**16**	1	0	0	0	0	0	0	0	1	0	1	0	3
**17**	1	1	0	1	1	1	1	1	1	0	1	1	4
**18**	1	1	0	1	0	0	0	1	1	0	1	1	4
**19**	0	0	0	1	0	0	0	1	1	0	1	1	3
**20**	0	0	0	0	0	0	0	0	0	0	0	0	0
**21**	1	1	0	0	0	0	0	1	0	1	1	1	3
**22**	0	0	0	0	0	0	0	1	0	0	0	0	1
**23**	0	0	0	0	0	0	0	0	0	0	0	0	0
**24**	0	0	0	0	0	0	0	0	0	0	0	0	0
**25**	0	1	0	0	0	0	0	0	0	1	0	0	2
**26**	0	0	0	0	0	0	0	0	0	0	0	0	0
**27**	0	0	0	0	0	0	0	0	0	0	0	0	0
**28**	1	1	0	1	0	1	1	1	1	0	1	1	4
**29**	1	0	0	0	0	0	0	0	0	0	1	1	2
**30**	1	1	0	0	0	1	1	0	0	0	1	1	4
**31**	0	0	0	0	0	0	0	0	0	0	0	0	0
**32**	1	1	0	1	1	1	1	1	1	0	1	1	4
**33**	1	1	1	1	1	1	1	1	1	0	1	1	4
**34**	1	1	0	1	1	1	1	1	1	0	1	1	4
**35**	0	1	0	0	0	1	1	0	0	0	0	0	3
**36**	0	1	0	1	1	1	1	1	1	0	1	1	4
**37**	1	0	0	0	0	0	0	0	0	0	0	0	1
**38**	0	0	0	0	0	0	0	0	0	0	0	0	0
**39**	0	0	0	0	0	0	0	0	0	0	0	0	0
**40**	0	1	0	0	0	0	0	0	0	0	0	0	1
**41**	0	0	0	0	0	0	0	0	0	1	0	0	1
**42**	0	1	0	0	1	1	1	0	0	0	0	0	3
**43**	1	1	0	1	1	1	1	1	1	0	1	1	4
**44**	0	0	0	0	0	0	0	0	0	0	0	0	0
**45**	0	1	0	0	0	1	1	1	0	0	0	1	4

S2. www.phage-therapy.org/calculators/cocktail_optimizer.html [return to Table of Contents]

[1] Inclusion of a bacterium within the host range of at least one phage that is found in a single cross-resistance group counts there as a depth of 1. Activity depth per bacterium in the overall analysis can be up to 4, corresponding to the four cross-resistance groups. Whether or not a bacterium is hit by at least one phage that is found in each cross-resistance group (1 or 0, respectively) is then added across rows to generate the last column, indicating depth of activity per bacterium. Thus, for the first row, 0 + 1 + 1+ 1 = 3. That procedure is equivalent to as illustrated in Equations 3 and 4 as well as Figure 6 as presented in the main article.

```
<!DOCTYPE html PUBLIC "-//W3C//DTD XHTML 1.0 Transitional//EN" "http://www.w3.org/TR/xhtml1/DTD/xhtml1-tran-
sitional.dtd">
<html xmlns="http://www.w3.org/1999/xhtml">
<head><meta charset="iso-8859-1">

<title>Phage Cocktail Optimizer - Bacteriophage Ecology Group</title>

<meta property="og:image" content="/images/logo.png"/>

<META name="description" content= "Phage Cocktail Optimizer | What combination of phages will most broadly
impact your favorite bacterial species? |  " />

<META name="keywords" content=

Stephen T. Abedon,
Stephen Abedon,
Steve Abedon,
S. T. Abedon,
S.T. Abedon,
S. Abedon,
ST Abedon,
S Abedon,
SAbedon,
Abedon,
Cocktail,
Phage Cocktail,
Phage Cocktail Optimizer,
Spot Test,
Spot Assay,
Host Range,
Maximize Host Range,
Phage Host Range,
Spectrum of Activity,
Cocktail Spectrum of Activity,
Cocktail Host Range

>

<link href="http://www.BiologyAsPoetry.com/css/terms_import_BaP.css" rel="stylesheet" type="text/css" />
<link href="http://www.BiologyAsPoetry.com/css/forms.css" rel="stylesheet" type="text/css" />
<link rel="icon" href="/favicon.ico" />
```

```
<script type="text/javascript" src="http://www.BiologyAsPoetry.com/scripts/other_links.js"></script>
<script type="text/javascript" src="http://www.BiologyAsPoetry.com/scripts/front_material.js"> </script>

<script type="text/javascript">

  var _gaq = _gaq || [];
  _gaq.push(['_setAccount', 'UA-23749643-1']);
  _gaq.push(['_trackPageview']);

  (function() {
    var ga = document.createElement('script'); ga.type = 'text/javascript'; ga.async = true;
    ga.src = ('https:' == document.location.protocol ? 'https://ssl' : 'http://www') + '.google-analyt-
ics.com/ga.js';
    var s = document.getElementsByTagName('script')[0]; s.parentNode.insertBefore(ga, s);
  })();

</script>

</head>

<body>

<div id="fb-root"></div>
<script>(function(d, s, id) {
  var js, fjs = d.getElementsByTagName(s)[0];
  if (d.getElementById(id)) return;
  js = d.createElement(s); js.id = id;
  js.src = "//connect.facebook.net/en_US/sdk.js#xfbml=1&version=v2.0";
  fjs.parentNode.insertBefore(js, fjs);
}(document, 'script', 'facebook-jssdk'));</script>

<script type="text/javascript" src="http://www.google.com/jsapi"></script>

<script type="text/javascript"> IsPrivate(); </script> <script type="text/javascript"> front_mate-
rial("phage_therapy","Phage Cocktail Optimizer","choose what phages will maximize your cocktail's spectrum of
activity","F","F","F"); </script>

<H1 id="TitleTransparent"><font color=transparent>Phage Cocktail Host-Range Optimizer</font></H1>

</P><P id="DefinitionCenter"><font color=#A0A0A0 size=2>&infin; generated and posted on
2020.10.22 &infin;</font></P>
```

```
</P><P id="DefinitionCenterEmphasis">What combination of phages will most broadly impact your favorite bacte-
rial species?

<script type="text/javascript"> other_links("TX","Phage Cocktail Optimizer","F","F","F","F","BEG","F");
</script>

<! /////////////////////////////////////////////////////////////////////////////////////////>

<! Place Dropdown box here >

<BR><BR>

<form name="nav">
<select id="listbox_positioning_phagetherapyorg" name="SelectURL" onchange="document.location.href=docu-
ment.nav.SelectURL.options[document.nav.SelectURL.selectedIndex].value">
  <option value="index.html">Quick Navigation of Phage-Therapy.org</option>
  <option value="http://phage-therapy.org/writings/bacteriophages.html">What are Phages as well as Phage
Therapy?</option>
  <option value="http://publications.phage-therapy.org">Phage Therapy Publications</option>
  <option value="videos.html">Phage Therapy Videos</option>
  <option value="advantages.html">Advantages of Phage Therapy</option>
  <option value="http://www.phage-therapy.org/virtual_book.html">Phage Therapy Virtual "Book"</option>
  <option value="http://companies.phage.org">Phage Companies</option>Killing Titer Calculator
  <option value="http://www.phage.org">Phage.org (Bacteriophage Ecology Group)</option>
  <option value="http://www.phage-therapy.org/resources.html">Summary of Content/Pages/Links</option>
  <option value="http://www.phage-therapy.org">Phage-Therapy.org Main Page</option>
</select>
</form>

<! /////////////////////////////////////////////////////////////////////////////////////////>


</P><P id="DefinitionCenter"><font color=red>Please cite as:</font><BR><BR><font color=#8A5C00><B>Stephen T.
Abedon</B><BR>Phage Cocktail Optimizer<BR><a onclick="parent.location = 'http://www.phage-therapy.org/calcu-
lators/cocktail_optimizer.html'"><u>phage-therapy.org/calculators/cocktail_opti-
mizer.html</u></a></font><BR><BR>

</P><div id="poetry_definition_positioning"><table id="poetry_definition" cellspacing="2"><tr><td class="po-
etry_definition_text"><font color=red>The idea behind this page is that you have data on the host range of a
number of phages and this information could/should be included in phage cocktail design, but now what? Well,
one answer is that you could figure out by eye or by 'hand', or using Excel, what combination of phages pro-
vides the broadest overall spectrum of activity&hellip; Or you could use this app!<BR><BR>A 'postive' (1) is
```

a phage that has a given bacterium within its host range, however host range might be defined. A 'negative' (0) refers to a phage whose host range does not overlap the bacterium in question. It is up to you to figure out what sort of result should constitute a 1 versus a 0. Sorry, this app deals with binary results only.

Often host range is determined using either spot tests or efficeincy of plating (EOP) determinations.

Keep in mind when doing spot tests that only clear(ish) spots should be counted as positives. It is fine to have resistant bacterial colonies growing within the spot. It is not OK, however, to count spots that are only a bit less turbid than the untreated lawn as positives, and certainly do not count spots that display only isolated plaques, no matter how many plaques you might be able to count (if you can count 'em, then there are not enought of 'em to call a 'spot' a positive).

That is, should a spot have been initiated using, e.g., 10⁴ to 10⁶ plaque-forming units, then your still being able to see plaques – unless those plaques are nearly microscopic ('pin prick plaques') – would be an indication of an expesically low EOP… Do the math!

If you are using instead EOP determinations, then you will need to have some sort of minimum EOP cuttoff, e.g., perhaps 10⁻¹? Lower? I really can't tell you, but only that somehow positive values, whatever they are, will need to be counted as 1s and negatives as 0s.

Thus, your goal will be to generate one one-dimensional array (i.e., a row) for each potential target baterial strain tested, where that array consists of ones and zeros, with each digit, whether positive or negative, referring to a single tested phage type.

An example could be 110111000000, which would represent one tested bacterial strain and 14 different tested phages. The length of this array thus will be that of the numbers of phages tested, i.e., in the example, 14 phages

Note: Keep phage data order, as represented by 1s and 0s, consistent from row-to-row, that is, phage 1, then phage 2, then phage 3 should always be phage 1 then 2 then 3, and never instead phage 1 then 3 then 2 when going from row to row.

The number of rows, then, will be the number bacteria tested, though what order rows are provided in shouldn't matter, just so long as you know what bacterial strain is associated with what row. Thus, rows represent the data for a single bacterium and columns represent the data for a single phage.

Don't include in these analyses non-target hosts, e.g., <I>E. coli</I> negatives should not be included when you are targetting <I>P. aeruginosa</I>. The goal of a cocktail afterall is to 'hit' target bacteria and not 'hit' non-target bacteria.

Note that it is possible to optimize not just for cocktail breadth of spectrum of activity but also for cocktail 'depth' of spectrum of activity, that is, the number of phages impacting each targeted bacterium (depth) versus the number of bacteria impacted by a cocktail (breadth). By default this is 1 or more phages/bacerium (≥ 0), meaning that optimization will be for breadth only. Number of phages impacting each bacterium also can be set to greater than 1, e.g., ≥ 2 or ≥ 3, however, thus optimizing for depth of spectrum of activity as well. Depth of spectrum of activity is important toward minimizing bacterial evolution of phage resistance, which by definition will require at least two phages negatively impacting each targeted bacterial strain, though whether included phages will actually reduce the potential for bacterial evolution of phage resistance for every bacterial strain targeted, meaning minimal potential for cross resistrance, will still need to be determined experimentally.

A caveat is that depth of activity should be defined in terms of different phage cross-resistance groups since if a bacterium can easily mutant to simultaneous resistance to two different phages, then those two phages together will not have a substantial impact on the potential for a bacterium to mutate to phage resistance. For how to deal with an occurence of cross resistance to different phages making up a phage cocktail, see http://www.phage-therapy.org/calculators/xresistance_avoider.html.

The

```
following is a test array to copy and paste in if you just want to try the app out, and this should be iden-
tical to the placeholder found in the text area, below:<BR><BR><font face="Courier New"
color=black>01010110000100010110000000000000000000000000000000<BR>0001000110010100000001000000000000000000000
0000000<BR>00001100000010010000001000100010000000000000000000<BR>00010011010000001000000000000100000000110000
000000<BR>00000000000010000000000000000000001000000001100000<BR>000000110101000001010001000000000000000000000
00000<BR>00100000000010000000000011001000000000000000000000<BR>0000010001001100001000000000000000000000000001
0000<BR>00000000000010000000001001000000000100000000000000<BR>11000000000010100000000000000000110001000000000
000<BR>00000000000010011000000100000000000000000000000000<BR>011000100000110000000001000000000000000000000000
00<BR>00000001000000001000010000000100000000000000101000<BR>0001000000000000000000100000000101000000000100000
0<BR>00000000100001000010000000000000000000100000000000<BR>10011011000000000010000000001001001000000000000000
<BR>00000001000100001001010000000000010000000000000000<BR>00000000000001000001000010011000000000000000000000<
BR>10011001000111000001000000000000000010000000000000<BR>01000000000000000000000000000010000000000000000000<B
R>00100011000000000000000010000000100000000000000000<BR>10110011001000000000100000000100001100000000000000<BR
>00000110000100000100001000000000101000000000000000<BR>10000001000000100000011110000000000000000000000000<BR>
00001110010000000001000000101000000000000000000000</font><BR><BR>Note, regardless, that only numbers, and
line breaks, should be entered into the various input boxes found below. Also, you can't really paste di-
rectly from Excel but insted have to paste first into Word and then remove the tab (^t) characters via a
global search and replace. Lastly, be careful when you copy and paste from Word as you are liable to carry
over a break line at the end of the list, which I haven't yet figured out how to deal with. :-
/</td></tr></table></div>

<a name="calculator">

</P><div id="facebookpositionmainpagecenter"><div class="fb-like" data-href="http://Phage-Therapy.org/calcu-
lators/cocktail_optimizer.html" data-layout="standard" data-action="like" data-show-faces="true" data-
share="true"></div></div>

</a>

</P><P id="Definition"><!BR><!BR></P>

  <div class='wrapper'>
    <BR>
    <form id='nameForm'>
    <div class='form-uname'>
        <label><font color=blue><B>Optimize Cocktail Spectrum of Activity</B></label><BR><BR>

        <label id='numbersof' for='numbersofField'><B>Enter the number of phages and hosts tested:</B></la-
bel>

        <BR><BR>
```

```
<label id='numbersofphages' for='numbersofphagesField'>Number of phages tested =  </label>
<input id='numbersofphagesField' type='number' maxlength='50' value="16"></input>
<BR><font size=3 color=red>Above is the <I>length</I> of your rows of 1s and 0s.</font>
<BR><font size=3 color=red>The more phages, up to 50, the slower the output,</font>
<BR><font size=3 color=red>so best to first winnow down your candidate phages by eye!</font>

<BR><BR>

<label id='numbersofbacteria' for='numbersofbacteriaField'>Number of bacteria tested =  </label>
<input id='numbersofbacteriaField' type='number' maxlength='50' value="25"></input>
<BR><font size=3 color=red>Above is the <I>number</I> of your rows of 1s and 0s.</font>

<BR><BR>

<label id='TwoDarray' for='TwoDarrayField'><B>Enter 2D Array of Host Range Results:</B></label>

<BR><BR>

<textarea id="TwoDarrayField" name="subject" placeholder="1=pos pheno, rows=hosts, columns (dig-
its)=phages:         0101011000010001011000000000000000000000000000000
0001000110010100000001000000000000000000000000000 0000110000001001000000100010001000000000000000000
0001001101000000100000000000001000000001100000000000 0000000000001000000000000000000000100000000110000
0000000110101000001010001000000000000000000000000 0010000000001000000000001100100000000000000000000
0000010001001100001000000000000000000000000010000 0000000000001000000000100100000000100000000000000
1100000000001010000000000000001100010000000000000 0000000000001001100000010000000000000000000000000
0110001000001100000000010000000000000000000000000 0000000100000000100001000000010000000000000000101000
0001000000000000000001000000000101000000000001000000 0000000010000100001000000000000000000001000000000000
1001101100000000001000000000100100100000000000000 0000000100010000100101000000000001000000000000000
0000000000001000001000010011000000000000000000000 1001100100011100000100000000000000001000000000000000
0100000000000000000000000000001000000000000000000 0010001100000000000000010000000100000000000000000
1011001100100000000010000000010000110000000000000 0000011000010000010000100000000010100000000000000
1000000100000010000001111000000000000000000000000 0000111001000000000100000010100000000000000000000"
rows="15" cols="60"></textarea>

<BR><!BR>

<BR><font size=3 color=red>1 means pos result, e.g., non-turbid spot (some colonies OK).</font>
<BR><font size=3 color=red>0 means neg result, e.g., turbid spot or individual plaques.</font>
<BR><font size=3 color=red>010 means first phage is neg, second is pos, third is neg&hellip;</font>
<BR><font size=3 color=red>Start a new row to enter data from an additional host.</font>
<BR><font size=3 color=red>Make sure that each row is separated by a break line.</font>
<BR><font size=3 color=red>For now, keep phage #s below 101 and host #s below 501.</font>
```

```
        <! Is there really any reason to place the above limits on optimizations? >

        <BR><BR>

        <label id='numbersof' for='numbersofField'><B>Enter some additional parameters:</B></label>

        <BR><BR>

        <label id='maxsizecocktail' for='maxsizecocktailField'>Number of phages/cocktail =  </label>
        <input id='maxsizecocktailField' type='number' maxlength='50' value="4"></input>
        <BR><font size=3 color=red>Above is the number of phage types in the cocktail.</font>
        <BR><font size=3 color=red>The larger your cocktail size, the slower the output.</font>
        <BR><font size=3 color=red>Note: too many phages per can be agonizingly slow&hellip;</font>

        <BR><BR>

        <label id='numberofhits' for='numberofhitsField'>Depth of cocktail activity &ge;  </label>
        <input id='numberofhitsField' type='number' maxlength='50' value="1"></input>
        <BR><font size=3 color=red>Above is the number of phages impacting each bacterium.</font>
        <BR><font size=3 color=red>For cocktail breadth you want at least 1 phage/bacterium.</font>
        <BR><font size=3 color=red>For cocktail depth you want at least 2 phages/bacterium.</font>

        <BR><BR>

        <label id='maxnumbershown' for='maxnumbershownField'>Maximum cocktails shown =  </label>
        <input id='maxnumbershownField' type='number' maxlength='50' value="50"></input>
        <BR><font size=3 color=red>Of many possible combinations, show only the top (above) number.</font>

     </div>
 <BR>
   <div class='form-sub'>
        <button id='subButton1' type='button'>Optimize Cocktail Spectrum of Activity</button>
        <button type="reset" value="Reset">Reset Form</button>
     </div>
</form>

<! Code is based on that found here: http://codepen.io/NickersF/pen/EVjVNd >

<div>
    <p id='result0'></p></div>
<div>
    <p id='result1'></p></div>
```

```
<div>
    <p id='result2'></p></div>
<div>
    <p id='result3'></p></div>
<div>
    <p id='result4'></p></div>
<div>
    <p id='result5'></p></div>
<div>
    <p id='result6'></p></div>
<div>
    <p id='result7'></p></div>
<div>
    <p id='result8'></p></div>
<div>
    <p id='result9'></p></div>
<div>
    <p id='result10'></p></div>
<div>
    <p id='result11'></p></div>
<div>
    <p id='result12'></p></div>
<div>
    <p id='result13'></p></div>
<div>
    <p id='result14'></p></div>
<div>
    <p id='result15'></p></div>
<div>
    <p id='result16'></p></div>
<div>
    <p id='result17'></p></div>
<div>
    <p id='result18'></p></div>
<div>
    <p id='result19'></p></div>
<div>
    <p id='result20'></p></div>
<div>
    <p id='result21'></p></div>
<div>
    <p id='result22'></p></div>
```

```
<div>
    <p id='result23'></p></div>
<div>
    <p id='result24'></p></div>
<div>
    <p id='result25'></p></div>
<div>
    <p id='result26'></p></div>
<div>
    <p id='result27'></p></div>
<div>
    <p id='result28'></p></div>
<div>
    <p id='result29'></p></div>
<div>
    <p id='result30'></p></div>
<div>
    <p id='result31'></p></div>
<div>
    <p id='result32'></p></div>
<div>
    <p id='result33'></p></div>
<div>
    <p id='result34'></p></div>
<div>
    <p id='result35'></p></div>
<div>
    <p id='result36'></p></div>
<div>
    <p id='result37'></p></div>
<div>
    <p id='result38'></p></div>
<div>
    <p id='result39'></p></div>
<div>
    <p id='result40'></p></div>
<div>
    <p id='result41'></p></div>
<div>
    <p id='result42'></p></div>
<div>
    <p id='result43'></p></div>
```

```
<div>
    <p id='result44'></p></div>
<div>
    <p id='result45'></p></div>
<div>
    <p id='result46'></p></div>
<div>
    <p id='result47'></p></div>
<div>
    <p id='result48'></p></div>
<div>
    <p id='result49'></p></div>
<div>
    <p id='result50'></p></div>
<div>
    <p id='result51'></p></div>
<div>
    <p id='result52'></p></div>
<div>
    <p id='result53'></p></div>
<div>
    <p id='result54'></p></div>
<div>
    <p id='result55'></p></div>
<div>
    <p id='result56'></p></div>
<div>
    <p id='result57'></p></div>
<div>
    <p id='result58'></p></div>
<div>
    <p id='result59'></p></div>
<div>
    <p id='result60'></p></div>
<div>
    <p id='result61'></p></div>

    </div>

    <script src="http://www.phage-therapy.org/scripts/cocktail_optimizer.js"></script>
```

```
</P>



</P><P id="Additional"><BR><BR>

<iframe src="http://www.facebook.com/plugins/like.php?href=http://www.phage-therapy.org/calculators/cock-
tail_optimizer.html"
        scrolling="no" frameborder="0"
        style="border:none; width:450px; height:24px"></iframe>

</P>

<div id="googlesearchpositionalt">

<script>
  (function() {
    var cx = '018033303197021436553:1odoahemmfs';
    var gcse = document.createElement('script');
    gcse.type = 'text/javascript';
    gcse.async = true;
    gcse.src = 'https://cse.google.com/cse.js?cx=' + cx;
    var s = document.getElementsByTagName('script')[0];
    s.parentNode.insertBefore(gcse, s);
  })();
</script>
<gcse:search></gcse:search>

</div>

<script type="text/javascript"> other_links("BX","Phage Cocktail Optimizer","F","F","F","F","BEG","F");
</script>

</P>

<script type="text/javascript" src="http://www.biologyaspoetry.com/scripts/end_materials.js"> </script>

</body>
</html>
```

S3. www.phage-therapy.org/scripts/cocktail_optimizer.js [return to Table of Contents]

```
// the function which handles the input field logic
function cocktail_optimizer() {

    var start = new Date();

    const maxphagenum = 100;
    const maxbacterianum = 500;
    const maxsizecoctail = 20;
    const maxtodisplay = 50;
    const numberofhitsconst = 2;  // zero for host-range breadth analysis

    var numbersofphagesField = document.getElementById('numbersofphagesField').value;
    var numbersofbacteriaField = document.getElementById('numbersofbacte-
riaField').value;
    var numberofhitsField = document.getElementById('numberofhitsField').value;
    var TwoDarrayField = document.getElementById('TwoDarrayField').value;
    var maxsizecocktailField = document.getElementById('maxsizecocktailField').value;
    var maxnumbershownField = document.getElementById('maxnumbershownField').value;

    var errorvar = 'F';
    var i = 0;
    var j = 0;
    var k = 0;
//    var teststring = '';

    result0.textContent = '------------------------------------------------------------
---------';

////////////////////////////////////////////////////////////////////////////////
// INPUT ERROR CHECKING

    if (numbersofphagesField.length < 1 || numbersofphagesField <= 2 || numbersofphag-
esField > maxphagenum) {

    errorvar = 'T';
    result1.textContent = 'number of phages must be > 2 and < ' + (maxphagenum+1);
    result2.textContent = '------------------------------------------------------------
---------';

    }
```

```
    if ((numbersofbacteriaField.length < 1 || numbersofbacteriaField <= 2 || numbersof-
bacteriaField > maxbacterianum) && (errorvar == 'F')) {

    errorvar = 'T';
    result1.textContent = 'number of bacteria must be > 2 and < ' + (maxbacterianum+1);
    result2.textContent = '------------------------------------------------------------
---------';

    }

    if ((maxsizecocktailField.length < 1 || maxsizecocktailField <= 1 || maxsizecock-
tailField > maxsizecoctail) && (errorvar == 'F')) {

    errorvar = 'T';
    result1.textContent = 'proposed cocktails must be > 1 but < ' + maxsizecoctail + '
(sorry)';
    result2.textContent = '------------------------------------------------------------
---------';

    }

    if ((maxsizecocktailField.length < 1 || maxsizecocktailField > numbersofphagesField
- 2) && (errorvar == 'F')) {

    errorvar = 'T';
    result1.textContent = 'cocktail size must be < number of phages tested - 1';
    result2.textContent = '------------------------------------------------------------
---------';

    }

    if ((numberofhitsField.length < 1 || numberofhitsField < 1 || numberofhitsField >
maxsizecocktailField) && (errorvar == 'F')) {

    errorvar = 'T';
    result1.textContent = 'Each bacterium \'hit\' by < 1 or > cocktail size phages';
    result2.textContent = '------------------------------------------------------------
---------';

    }
    else
    {
```

```
    numberofhitsField = numberofhitsField - 1;
    }



    if ((maxnumbershownField.length < 1 || maxnumbershownField <= 2 || maxnumbershown-
Field > maxtodisplay) && (errorvar == 'F')) {

    errorvar = 'T';
    result1.textContent = 'only the top ' + maxtodisplay + ' results will be shown
(sorry)';
    result2.textContent = '------------------------------------------------------------
---------';

    }

    if ((TwoDarrayField.length < 1) && (errorvar == 'F')) {

    errorvar = 'T';
    result1.textContent = 'you must enter a 2D array to run this app';
    result2.textContent = '------------------------------------------------------------
---------';

    }

    else {

    var numberrows = new Array(numbersofbacteriaField);
    for (i = 0; i < numberrows.length; i++) {
        numberrows[i] = 0;
    }

//    var bacteriaarray = new Array(numbersofphagesField);
//    for (j = 0; j < bacteriaarray.length; j++) {
//        bacteriaarray[j] = 0;
//    }
//
//    bacteriaarrayinit = bacteriaarray;

    var TwoDArray = new Array(numbersofbacteriaField);
    for (k = 0; k < numbersofbacteriaField; k++) {
        TwoDArray[k] = new Array(numbersofphagesField);
    }
```

```
    for (k = 0; k < TwoDArray.length; k++) {
        for (j = 0; j < numbersofphagesField; j++) {
    TwoDArray[k][j] = 0;
    } }


    k = 0;
    for (i = 0; i < TwoDarrayField.length; i++) {
    if (TwoDarrayField.charCodeAt(i) == 10) {
        k = k + 1
    } }

    result1.textContent = '';
    result2.textContent = '';
    result3.textContent = '';

    if (errorvar == 'F' && numbersofbacteriaField != k+1) {

    errorvar = 'T';
    result1.textContent = '# of bacteria being tested (' + numbersofbacteriaField + ')
must = # rows (' + (k+1) + ')';
    result2.textContent = 'or you might have a carriage return hanging off end';
    result3.textContent = '-----------------------------------------------------------
---------';
//    result4.textContent = TwoDarrayField.charCodeAt(TwoDarrayField.length-1);
        }

    if (errorvar == 'F') {

    j = 0;
    k = 0;

    for (i = 0; i < TwoDarrayField.length; i++) {

    var localchar = TwoDarrayField.charAt(i);

    if ((localchar == 0 || localchar == 1) && (localchar != ' ')) {
//    if (localchar == 0 || localchar == 1) {

    if (TwoDarrayField.charCodeAt(i) != 10) {
```

```
//    bacteriaarray[j] = localchar;

    TwoDArray[k][j] = Number(localchar);

    j = j + 1;
//    teststring = teststring.concat(localchar);

    }

    if (TwoDarrayField.charCodeAt(i) == 10) {

    numberrows[k] = j;
    j = 0;
    k = k + 1;
    }
    }

    else {

    errorvar = 'T';
    result1.textContent = 'only 1s and 0s are allowed in the 2D array';
    result2.textContent = '-----------------------------------------------------------
---------';

    }

    }

    numberrows[k] = j;
    k = k + 1;

    } }

    if (errorvar == 'F') {

    var rowonenum = numberrows[0];

    for (i = 0; i < numbersofbacteriaField; i++) {

    if (numberrows[i] != rowonenum) {

    errorvar = 'S';
```

```
    }

    if (errorvar == 'S' && numbersofbacteriaField == k) {

    errorvar = 'T';
    result1.textContent = 'rows must be same length, phage #s consistent';
    result2.textContent = '-----------------------------------------------------------
---------';

    }

    } }

///////////////////////////////////////////////////////////////////////////////
// ACTUAL CALCULATIONS

if (errorvar == 'F') {

var j0, j1, j2, j3, j4, j5, j6, j7, j8, j9, j10, j11, j12, j13, j14, j15, j16, j17,
j18, j19, j20 = 0;
var numbacthit = 0;
var numcocktails = 0;

    var cocktailhits = new Array(maxnumbershownField);
    for (i = 0; i < maxnumbershownField; i++) {
        cocktailhits[i] = new Array(maxsizecocktailField+1); // score, phage #1, phage
#2...
    }

    for (i = 0; i < maxnumbershownField; i++) {
        for (j = 0; j < maxsizecocktailField+1; j++) {
    cocktailhits[i][j] = 0;
    } }

//---------------------------------------------------//

if (maxsizecocktailField == 2) {

    for (j0 = 0; j0 < numbersofphagesField-1; j0++) {
    for (j1 = j0+1; j1 < numbersofphagesField; j1++) {
```

```
    cocktailhits.sort(function(a, b) {
        return a[0] - b[0];
    });

    if (j1 > j0) {
    numbacthit = 0;
    for (k = 0; k < numbersofbacteriaField; k++) {

        if (TwoDArray[k][j0] + TwoDArray[k][j1] > numberofhitsField) {
            numbacthit = numbacthit + 1;
            numcocktails = numcocktails + 1;
        }
    }

    for (i = 0; i < maxnumbershownField; i++) {
        if (numbacthit > cocktailhits[i][0]) {
            cocktailhits[i][0] = numbacthit;
            cocktailhits[i][1] = j0 + 1;
            cocktailhits[i][2] = j1 + 1;
            i = maxnumbershownField;
        }
    }
    }

    }}}

//---------------------------------------------------//

if (maxsizecocktailField == 3) {

    for (j0 = 0; j0 < numbersofphagesField-2; j0++) {
    for (j1 = j0+1; j1 < numbersofphagesField-1; j1++) {
    for (j2 = j1+1; j2 < numbersofphagesField; j2++) {

    cocktailhits.sort(function(a, b) {
        return a[0] - b[0];
    });

    if (j2 > j1 && j1 > j0) {
    numbacthit = 0;
    for (k = 0; k < numbersofbacteriaField; k++) {
```

```
        if (TwoDArray[k][j0] + TwoDArray[k][j1] + TwoDArray[k][j2] > numberofhitsField)
{
            numbacthit = numbacthit + 1;
            numcocktails = numcocktails + 1;
        }
    }

    for (i = 0; i < maxnumbershownField; i++) {
        if (numbacthit > cocktailhits[i][0]) {
            cocktailhits[i][0] = numbacthit;
            cocktailhits[i][1] = j0 + 1;
            cocktailhits[i][2] = j1 + 1;
            cocktailhits[i][3] = j2 + 1;
            i = maxnumbershownField;
        }
    }
    }

    }}}}

//----------------------------------------------------//

if (maxsizecocktailField == 4) {

    for (j0 = 0; j0 < numbersofphagesField-3; j0++) {
    for (j1 = j0+1; j1 < numbersofphagesField-2; j1++) {
    for (j2 = j1+1; j2 < numbersofphagesField-1; j2++) {
    for (j3 = j2+1; j3 < numbersofphagesField; j3++) {

    cocktailhits.sort(function(a, b) {
        return a[0] - b[0];
    });

    if (j3 > j2 && j2 > j1 && j1 > j0) {
    numbacthit = 0;
    for (k = 0; k < numbersofbacteriaField; k++) {

        if (TwoDArray[k][j0] + TwoDArray[k][j1] + TwoDArray[k][j2] + TwoDArray[k][j3] >
numberofhitsField) {
            numbacthit = numbacthit + 1;
            numcocktails = numcocktails + 1;
        }
```

```
    }

    for (i = 0; i < maxnumbershownField; i++) {
        if (numbacthit > cocktailhits[i][0]) {
            cocktailhits[i][0] = numbacthit;
            cocktailhits[i][1] = j0 + 1;
            cocktailhits[i][2] = j1 + 1;
            cocktailhits[i][3] = j2 + 1;
            cocktailhits[i][4] = j3 + 1;
            i = maxnumbershownField;
        }
    }
    }

    }}}}}

//---------------------------------------------------//

if (maxsizecocktailField == 5) {

    for (j0 = 0; j0 < numbersofphagesField-4; j0++) {
    for (j1 = j0+1; j1 < numbersofphagesField-3; j1++) {
    for (j2 = j1+1; j2 < numbersofphagesField-2; j2++) {
    for (j3 = j2+1; j3 < numbersofphagesField-1; j3++) {
    for (j4 = j3+1; j4 < numbersofphagesField; j4++) {

    cocktailhits.sort(function(a, b) {
        return a[0] - b[0];
    });

    if (j4 > j3 && j3 > j2 && j2 > j1 && j1 > j0) {
    numbacthit = 0;
    for (k = 0; k < numbersofbacteriaField; k++) {

        if (TwoDArray[k][j0] + TwoDArray[k][j1] + TwoDArray[k][j2] + TwoDArray[k][j3] +
TwoDArray[k][j4] > numberofhitsField) {
            numbacthit = numbacthit + 1;
            numcocktails = numcocktails + 1;
        }
    }

    for (i = 0; i < maxnumbershownField; i++) {
```

```
        if (numbacthit > cocktailhits[i][0]) {
            cocktailhits[i][0] = numbacthit;
            cocktailhits[i][1] = j0 + 1;
            cocktailhits[i][2] = j1 + 1;
            cocktailhits[i][3] = j2 + 1;
            cocktailhits[i][4] = j3 + 1;
            cocktailhits[i][5] = j4 + 1;
            i = maxnumbershownField;
        }
    }
    }

    }}}}}}

//---------------------------------------------------//

if (maxsizecocktailField == 6) {

    for (j0 = 0; j0 < numbersofphagesField-5; j0++) {
    for (j1 = j0+1; j1 < numbersofphagesField-4; j1++) {
    for (j2 = j1+1; j2 < numbersofphagesField-3; j2++) {
    for (j3 = j2+1; j3 < numbersofphagesField-2; j3++) {
    for (j4 = j3+1; j4 < numbersofphagesField-1; j4++) {
    for (j5 = j4+1; j5 < numbersofphagesField; j5++) {

    cocktailhits.sort(function(a, b) {
        return a[0] - b[0];
    });

    if (j5 > j4 && j4 > j3 && j3 > j2 && j2 > j1 && j1 > j0) {
    numbacthit = 0;
    for (k = 0; k < numbersofbacteriaField; k++) {

        if (TwoDArray[k][j0] + TwoDArray[k][j1] + TwoDArray[k][j2] + TwoDArray[k][j3] +
TwoDArray[k][j4] + TwoDArray[k][j5] > numberofhitsField) {
            numbacthit = numbacthit + 1;
            numcocktails = numcocktails + 1;
        }
    }

    for (i = 0; i < maxnumbershownField; i++) {
        if (numbacthit > cocktailhits[i][0]) {
```

```
            cocktailhits[i][0] = numbacthit;
            cocktailhits[i][1] = j0 + 1;
            cocktailhits[i][2] = j1 + 1;
            cocktailhits[i][3] = j2 + 1;
            cocktailhits[i][4] = j3 + 1;
            cocktailhits[i][5] = j4 + 1;
            cocktailhits[i][6] = j5 + 1;
            i = maxnumbershownField;
        }
    }
    }

    }}}}}}}

//---------------------------------------------------//

if (maxsizecocktailField == 7) {

    for (j0 = 0; j0 < numbersofphagesField-6; j0++) {
    for (j1 = j0+1; j1 < numbersofphagesField-5; j1++) {
    for (j2 = j1+1; j2 < numbersofphagesField-4; j2++) {
    for (j3 = j2+1; j3 < numbersofphagesField-3; j3++) {
    for (j4 = j3+1; j4 < numbersofphagesField-2; j4++) {
    for (j5 = j4+1; j5 < numbersofphagesField-1; j5++) {
    for (j6 = j5+1; j6 < numbersofphagesField; j6++) {

    cocktailhits.sort(function(a, b) {
        return a[0] - b[0];
    });

    if (j6 > j5 && j5 > j4 && j4 > j3 && j3 > j2 && j2 > j1 && j1 > j0) {
    numbacthit = 0;
    for (k = 0; k < numbersofbacteriaField; k++) {

        if (TwoDArray[k][j0] + TwoDArray[k][j1] + TwoDArray[k][j2] + TwoDArray[k][j3] +
TwoDArray[k][j4] + TwoDArray[k][j5] + TwoDArray[k][j6] > numberofhitsField) {
            numbacthit = numbacthit + 1;
            numcocktails = numcocktails + 1;
        }
    }

    for (i = 0; i < maxnumbershownField; i++) {
```

```
        if (numbacthit > cocktailhits[i][0]) {
            cocktailhits[i][0] = numbacthit;
            cocktailhits[i][1] = j0 + 1;
            cocktailhits[i][2] = j1 + 1;
            cocktailhits[i][3] = j2 + 1;
            cocktailhits[i][4] = j3 + 1;
            cocktailhits[i][5] = j4 + 1;
            cocktailhits[i][6] = j5 + 1;
            cocktailhits[i][7] = j6 + 1;
            i = maxnumbershownField;
        }
    }
    }

    }}}}}}}}

//---------------------------------------------------//

if (maxsizecocktailField == 8) {

    for (j0 = 0; j0 < numbersofphagesField-7; j0++) {
    for (j1 = j0+1; j1 < numbersofphagesField-6; j1++) {
    for (j2 = j1+1; j2 < numbersofphagesField-5; j2++) {
    for (j3 = j2+1; j3 < numbersofphagesField-4; j3++) {
    for (j4 = j3+1; j4 < numbersofphagesField-3; j4++) {
    for (j5 = j4+1; j5 < numbersofphagesField-2; j5++) {
    for (j6 = j5+1; j6 < numbersofphagesField-1; j6++) {
    for (j7 = j6+1; j7 < numbersofphagesField; j7++) {

    cocktailhits.sort(function(a, b) {
        return a[0] - b[0];
    });

    if (j7 > j6 && j6 > j5 && j5 > j4 && j4 > j3 && j3 > j2 && j2 > j1 && j1 > j0) {
    numbacthit = 0;
    for (k = 0; k < numbersofbacteriaField; k++) {

        if (TwoDArray[k][j0] + TwoDArray[k][j1] + TwoDArray[k][j2] + TwoDArray[k][j3] +
TwoDArray[k][j4] + TwoDArray[k][j5] + TwoDArray[k][j6] + TwoDArray[k][j7] > numberof-
hitsField) {
            numbacthit = numbacthit + 1;
            numcocktails = numcocktails + 1;
```

```
        }
    }

    for (i = 0; i < maxnumbershownField; i++) {
        if (numbacthit > cocktailhits[i][0]) {
            cocktailhits[i][0] = numbacthit;
            cocktailhits[i][1] = j0 + 1;
            cocktailhits[i][2] = j1 + 1;
            cocktailhits[i][3] = j2 + 1;
            cocktailhits[i][4] = j3 + 1;
            cocktailhits[i][5] = j4 + 1;
            cocktailhits[i][6] = j5 + 1;
            cocktailhits[i][7] = j6 + 1;
            cocktailhits[i][8] = j7 + 1;
            i = maxnumbershownField;
        }
    }
    }

    }}}}}}}}}

//---------------------------------------------------//

if (maxsizecocktailField == 9) {

    for (j0 = 0; j0 < numbersofphagesField-8; j0++) {
    for (j1 = j0+1; j1 < numbersofphagesField-7; j1++) {
    for (j2 = j1+1; j2 < numbersofphagesField-6; j2++) {
    for (j3 = j2+1; j3 < numbersofphagesField-5; j3++) {
    for (j4 = j3+1; j4 < numbersofphagesField-4; j4++) {
    for (j5 = j4+1; j5 < numbersofphagesField-3; j5++) {
    for (j6 = j5+1; j6 < numbersofphagesField-2; j6++) {
    for (j7 = j6+1; j7 < numbersofphagesField-1; j7++) {
    for (j8 = j7+1; j8 < numbersofphagesField; j8++) {

    cocktailhits.sort(function(a, b) {
        return a[0] - b[0];
    });

    if (j8 > j7 && j7 > j6 && j6 > j5 && j5 > j4 && j4 > j3 && j3 > j2 && j2 > j1 && j1
> j0) {
    numbacthit = 0;
```

```
    for (k = 0; k < numbersofbacteriaField; k++) {

        if (TwoDArray[k][j0] + TwoDArray[k][j1] + TwoDArray[k][j2] + TwoDArray[k][j3] +
TwoDArray[k][j4] + TwoDArray[k][j5] + TwoDArray[k][j6] + TwoDArray[k][j7] + Two-
DArray[k][j8] > numberofhitsField) {
            numbacthit = numbacthit + 1;
            numcocktails = numcocktails + 1;
        }
    }

    for (i = 0; i < maxnumbershownField; i++) {
        if (numbacthit > cocktailhits[i][0]) {
            cocktailhits[i][0] = numbacthit;
            cocktailhits[i][1] = j0 + 1;
            cocktailhits[i][2] = j1 + 1;
            cocktailhits[i][3] = j2 + 1;
            cocktailhits[i][4] = j3 + 1;
            cocktailhits[i][5] = j4 + 1;
            cocktailhits[i][6] = j5 + 1;
            cocktailhits[i][7] = j6 + 1;
            cocktailhits[i][8] = j7 + 1;
            cocktailhits[i][9] = j8 + 1;
            i = maxnumbershownField;
        }
    }
    }

    }}}}}}}}}}

//---------------------------------------------------//

if (maxsizecocktailField == 10) {

    for (j0 = 0; j0 < numbersofphagesField-9; j0++) {
    for (j1 = j0+1; j1 < numbersofphagesField-8; j1++) {
    for (j2 = j1+1; j2 < numbersofphagesField-7; j2++) {
    for (j3 = j2+1; j3 < numbersofphagesField-6; j3++) {
    for (j4 = j3+1; j4 < numbersofphagesField-5; j4++) {
    for (j5 = j4+1; j5 < numbersofphagesField-4; j5++) {
    for (j6 = j5+1; j6 < numbersofphagesField-3; j6++) {
    for (j7 = j6+1; j7 < numbersofphagesField-2; j7++) {
    for (j8 = j7+1; j8 < numbersofphagesField-1; j8++) {
```

```
    for (j9 = j8+1; j9 < numbersofphagesField; j9++) {

    cocktailhits.sort(function(a, b) {
        return a[0] - b[0];
    });

    if (j9 > j8 && j8 > j7 && j7 > j6 && j6 > j5 && j5 > j4 && j4 > j3 && j3 > j2 && j2
> j1 && j1 > j0) {
    numbacthit = 0;
    for (k = 0; k < numbersofbacteriaField; k++) {

        if (TwoDArray[k][j0] + TwoDArray[k][j1] + TwoDArray[k][j2] + TwoDArray[k][j3] +
TwoDArray[k][j4] + TwoDArray[k][j5] + TwoDArray[k][j6] + TwoDArray[k][j7] + Two-
DArray[k][j8] + TwoDArray[k][j9] > numberofhitsField) {
            numbacthit = numbacthit + 1;
            numcocktails = numcocktails + 1;
        }
    }

    for (i = 0; i < maxnumbershownField; i++) {
        if (numbacthit > cocktailhits[i][0]) {
            cocktailhits[i][0] = numbacthit;
            cocktailhits[i][1] = j0 + 1;
            cocktailhits[i][2] = j1 + 1;
            cocktailhits[i][3] = j2 + 1;
            cocktailhits[i][4] = j3 + 1;
            cocktailhits[i][5] = j4 + 1;
            cocktailhits[i][6] = j5 + 1;
            cocktailhits[i][7] = j6 + 1;
            cocktailhits[i][8] = j7 + 1;
            cocktailhits[i][9] = j8 + 1;
            cocktailhits[i][10] = j9 + 1;
            i = maxnumbershownField;
        }
    }
    }

    }}}}}}}}}}}

//----------------------------------------------------//

if (maxsizecocktailField == 11) {
```

```
    for (j0 = 0; j0 < numbersofphagesField-10; j0++) {
    for (j1 = j0+1; j1 < numbersofphagesField-9; j1++) {
    for (j2 = j1+1; j2 < numbersofphagesField-8; j2++) {
    for (j3 = j2+1; j3 < numbersofphagesField-7; j3++) {
    for (j4 = j3+1; j4 < numbersofphagesField-6; j4++) {
    for (j5 = j4+1; j5 < numbersofphagesField-5; j5++) {
    for (j6 = j5+1; j6 < numbersofphagesField-4; j6++) {
    for (j7 = j6+1; j7 < numbersofphagesField-3; j7++) {
    for (j8 = j7+1; j8 < numbersofphagesField-2; j8++) {
    for (j9 = j8+1; j9 < numbersofphagesField-1; j9++) {
    for (j10 = j9+1; j10 < numbersofphagesField; j10++) {

    cocktailhits.sort(function(a, b) {
        return a[0] - b[0];
    });

    if (j10 > j9 && j9 > j8 && j8 > j7 && j7 > j6 && j6 > j5 && j5 > j4 && j4 > j3 &&
j3 > j2 && j2 > j1 && j1 > j0) {
    numbacthit = 0;
    for (k = 0; k < numbersofbacteriaField; k++) {

        if (TwoDArray[k][j0] + TwoDArray[k][j1] + TwoDArray[k][j2] + TwoDArray[k][j3] +
TwoDArray[k][j4] + TwoDArray[k][j5] + TwoDArray[k][j6] + TwoDArray[k][j7] + Two-
DArray[k][j8] + TwoDArray[k][j9] + TwoDArray[k][j10] > numberofhitsField) {
            numbacthit = numbacthit + 1;
            numcocktails = numcocktails + 1;
        }
    }

    for (i = 0; i < maxnumbershownField; i++) {
        if (numbacthit > cocktailhits[i][0]) {
            cocktailhits[i][0] = numbacthit;
            cocktailhits[i][1] = j0 + 1;
            cocktailhits[i][2] = j1 + 1;
            cocktailhits[i][3] = j2 + 1;
            cocktailhits[i][4] = j3 + 1;
            cocktailhits[i][5] = j4 + 1;
            cocktailhits[i][6] = j5 + 1;
            cocktailhits[i][7] = j6 + 1;
            cocktailhits[i][8] = j7 + 1;
            cocktailhits[i][9] = j8 + 1;
```

```
            cocktailhits[i][10] = j9 + 1;
            cocktailhits[i][11] = j10 + 1;
            i = maxnumbershownField;
        }
    }
    }

    }}}}}}}}}}}}

//---------------------------------------------------//

if (maxsizecocktailField == 12) {

    for (j0 = 0; j0 < numbersofphagesField-11; j0++) {
    for (j1 = j0+1; j1 < numbersofphagesField-10; j1++) {
    for (j2 = j1+1; j2 < numbersofphagesField-9; j2++) {
    for (j3 = j2+1; j3 < numbersofphagesField-8; j3++) {
    for (j4 = j3+1; j4 < numbersofphagesField-7; j4++) {
    for (j5 = j4+1; j5 < numbersofphagesField-6; j5++) {
    for (j6 = j5+1; j6 < numbersofphagesField-5; j6++) {
    for (j7 = j6+1; j7 < numbersofphagesField-4; j7++) {
    for (j8 = j7+1; j8 < numbersofphagesField-3; j8++) {
    for (j9 = j8+1; j9 < numbersofphagesField-2; j9++) {
    for (j10 = j9+1; j10 < numbersofphagesField-1; j10++) {
    for (j11 = j10+1; j11 < numbersofphagesField; j11++) {

    cocktailhits.sort(function(a, b) {
        return a[0] - b[0];
    });

    if (j11 > j10 && j10 > j9 && j9 > j8 && j8 > j7 && j7 > j6 && j6 > j5 && j5 > j4 &&
j4 > j3 && j3 > j2 && j2 > j1 && j1 > j0) {
    numbacthit = 0;
    for (k = 0; k < numbersofbacteriaField; k++) {

        if (TwoDArray[k][j0] + TwoDArray[k][j1] + TwoDArray[k][j2] + TwoDArray[k][j3] +
TwoDArray[k][j4] + TwoDArray[k][j5] + TwoDArray[k][j6] + TwoDArray[k][j7] + Two-
DArray[k][j8] + TwoDArray[k][j9] + TwoDArray[k][j10] + TwoDArray[k][j11] > numberof-
hitsField) {
            numbacthit = numbacthit + 1;
            numcocktails = numcocktails + 1;
        }
```

```
    }

    for (i = 0; i < maxnumbershownField; i++) {
        if (numbacthit > cocktailhits[i][0]) {
            cocktailhits[i][0] = numbacthit;
            cocktailhits[i][1] = j0 + 1;
            cocktailhits[i][2] = j1 + 1;
            cocktailhits[i][3] = j2 + 1;
            cocktailhits[i][4] = j3 + 1;
            cocktailhits[i][5] = j4 + 1;
            cocktailhits[i][6] = j5 + 1;
            cocktailhits[i][7] = j6 + 1;
            cocktailhits[i][8] = j7 + 1;
            cocktailhits[i][9] = j8 + 1;
            cocktailhits[i][10] = j9 + 1;
            cocktailhits[i][11] = j10 + 1;
            cocktailhits[i][12] = j11 + 1;
            i = maxnumbershownField;
        }
    }
    }

    }}}}}}}}}}}}}

//---------------------------------------------------//

if (maxsizecocktailField == 13) {

    for (j0 = 0; j0 < numbersofphagesField-12; j0++) {
    for (j1 = j0+1; j1 < numbersofphagesField-11; j1++) {
    for (j2 = j1+1; j2 < numbersofphagesField-10; j2++) {
    for (j3 = j2+1; j3 < numbersofphagesField-9; j3++) {
    for (j4 = j3+1; j4 < numbersofphagesField-8; j4++) {
    for (j5 = j4+1; j5 < numbersofphagesField-7; j5++) {
    for (j6 = j5+1; j6 < numbersofphagesField-6; j6++) {
    for (j7 = j6+1; j7 < numbersofphagesField-5; j7++) {
    for (j8 = j7+1; j8 < numbersofphagesField-4; j8++) {
    for (j9 = j8+1; j9 < numbersofphagesField-3; j9++) {
    for (j10 = j9+1; j10 < numbersofphagesField-2; j10++) {
    for (j11 = j10+1; j11 < numbersofphagesField-1; j11++) {
    for (j12 = j11+1; j12 < numbersofphagesField; j12++) {
```

```
    cocktailhits.sort(function(a, b) {
        return a[0] - b[0];
    });

    if (j12 > j11 && j11 > j10 && j10 > j9 && j9 > j8 && j8 > j7 && j7 > j6 && j6 > j5
&& j5 > j4 && j4 > j3 && j3 > j2 && j2 > j1 && j1 > j0) {
    numbacthit = 0;
    for (k = 0; k < numbersofbacteriaField; k++) {

        if (TwoDArray[k][j0] + TwoDArray[k][j1] + TwoDArray[k][j2] + TwoDArray[k][j3] +
TwoDArray[k][j4] + TwoDArray[k][j5] + TwoDArray[k][j6] + TwoDArray[k][j7] + Two-
DArray[k][j8] + TwoDArray[k][j9] + TwoDArray[k][j10] + TwoDArray[k][j11] + Two-
DArray[k][j12] > numberofhitsField) {
            numbacthit = numbacthit + 1;
            numcocktails = numcocktails + 1;
        }
    }

    for (i = 0; i < maxnumbershownField; i++) {
        if (numbacthit > cocktailhits[i][0]) {
            cocktailhits[i][0] = numbacthit;
            cocktailhits[i][1] = j0 + 1;
            cocktailhits[i][2] = j1 + 1;
            cocktailhits[i][3] = j2 + 1;
            cocktailhits[i][4] = j3 + 1;
            cocktailhits[i][5] = j4 + 1;
            cocktailhits[i][6] = j5 + 1;
            cocktailhits[i][7] = j6 + 1;
            cocktailhits[i][8] = j7 + 1;
            cocktailhits[i][9] = j8 + 1;
            cocktailhits[i][10] = j9 + 1;
            cocktailhits[i][11] = j10 + 1;
            cocktailhits[i][12] = j11 + 1;
            cocktailhits[i][13] = j12 + 1;
            i = maxnumbershownField;
        }
    }
    }

    }}}}}}}}}}}}}

//---------------------------------------------------//
```

```
if (maxsizecocktailField == 14) {

    for (j0 = 0; j0 < numbersofphagesField-13; j0++) {
    for (j1 = j0+1; j1 < numbersofphagesField-12; j1++) {
    for (j2 = j1+1; j2 < numbersofphagesField-11; j2++) {
    for (j3 = j2+1; j3 < numbersofphagesField-10; j3++) {
    for (j4 = j3+1; j4 < numbersofphagesField-9; j4++) {
    for (j5 = j4+1; j5 < numbersofphagesField-8; j5++) {
    for (j6 = j5+1; j6 < numbersofphagesField-7; j6++) {
    for (j7 = j6+1; j7 < numbersofphagesField-6; j7++) {
    for (j8 = j7+1; j8 < numbersofphagesField-5; j8++) {
    for (j9 = j8+1; j9 < numbersofphagesField-4; j9++) {
    for (j10 = j9+1; j10 < numbersofphagesField-3; j10++) {
    for (j11 = j10+1; j11 < numbersofphagesField-2; j11++) {
    for (j12 = j11+1; j12 < numbersofphagesField-1; j12++) {
    for (j13 = j12+1; j13 < numbersofphagesField; j13++) {

    cocktailhits.sort(function(a, b) {
        return a[0] - b[0];
    });

    if (j13 > j12 && j12 > j11 && j11 > j10 && j10 > j9 && j9 > j8 && j8 > j7 && j7 >
j6 && j6 > j5 && j5 > j4 && j4 > j3 && j3 > j2 && j2 > j1 && j1 > j0) {
    numbacthit = 0;
    for (k = 0; k < numbersofbacteriaField; k++) {

        if (TwoDArray[k][j0] + TwoDArray[k][j1] + TwoDArray[k][j2] + TwoDArray[k][j3] +
TwoDArray[k][j4] + TwoDArray[k][j5] + TwoDArray[k][j6] + TwoDArray[k][j7] + Two-
DArray[k][j8] + TwoDArray[k][j9] + TwoDArray[k][j10] + TwoDArray[k][j11] + Two-
DArray[k][j12] + TwoDArray[k][j13] > numberofhitsField) {
            numbacthit = numbacthit + 1;
            numcocktails = numcocktails + 1;
        }
    }

    for (i = 0; i < maxnumbershownField; i++) {
        if (numbacthit > cocktailhits[i][0]) {
            cocktailhits[i][0] = numbacthit;
            cocktailhits[i][1] = j0 + 1;
            cocktailhits[i][2] = j1 + 1;
            cocktailhits[i][3] = j2 + 1;
```

```
            cocktailhits[i][4] = j3 + 1;
            cocktailhits[i][5] = j4 + 1;
            cocktailhits[i][6] = j5 + 1;
            cocktailhits[i][7] = j6 + 1;
            cocktailhits[i][8] = j7 + 1;
            cocktailhits[i][9] = j8 + 1;
            cocktailhits[i][10] = j9 + 1;
            cocktailhits[i][11] = j10 + 1;
            cocktailhits[i][12] = j11 + 1;
            cocktailhits[i][13] = j12 + 1;
            cocktailhits[i][14] = j13 + 1;
            i = maxnumbershownField;
        }
    }
    }

    }}}}}}}}}}}}}}}

//---------------------------------------------------//

if (maxsizecocktailField == 15) {

    for (j0 = 0; j0 < numbersofphagesField-14; j0++) {
    for (j1 = j0+1; j1 < numbersofphagesField-13; j1++) {
    for (j2 = j1+1; j2 < numbersofphagesField-12; j2++) {
    for (j3 = j2+1; j3 < numbersofphagesField-11; j3++) {
    for (j4 = j3+1; j4 < numbersofphagesField-10; j4++) {
    for (j5 = j4+1; j5 < numbersofphagesField-9; j5++) {
    for (j6 = j5+1; j6 < numbersofphagesField-8; j6++) {
    for (j7 = j6+1; j7 < numbersofphagesField-7; j7++) {
    for (j8 = j7+1; j8 < numbersofphagesField-6; j8++) {
    for (j9 = j8+1; j9 < numbersofphagesField-5; j9++) {
    for (j10 = j9+1; j10 < numbersofphagesField-4; j10++) {
    for (j11 = j10+1; j11 < numbersofphagesField-3; j11++) {
    for (j12 = j11+1; j12 < numbersofphagesField-2; j12++) {
    for (j13 = j12+1; j13 < numbersofphagesField-1; j13++) {
    for (j14 = j13+1; j14 < numbersofphagesField; j14++) {

    cocktailhits.sort(function(a, b) {
        return a[0] - b[0];
    });
```

```
    if (j14 > j13 && j13 > j12 && j12 > j11 && j11 > j10 && j10 > j9 && j9 > j8 && j8 >
j7 && j7 > j6 && j6 > j5 && j5 > j4 && j4 > j3 && j3 > j2 && j2 > j1 && j1 > j0) {
    numbacthit = 0;
    for (k = 0; k < numbersofbacteriaField; k++) {

        if (TwoDArray[k][j0] + TwoDArray[k][j1] + TwoDArray[k][j2] + TwoDArray[k][j3] +
TwoDArray[k][j4] + TwoDArray[k][j5] + TwoDArray[k][j6] + TwoDArray[k][j7] + Two-
DArray[k][j8] + TwoDArray[k][j9] + TwoDArray[k][j10] + TwoDArray[k][j11] + Two-
DArray[k][j12] + TwoDArray[k][j13] + TwoDArray[k][j14] > numberofhitsField) {
            numbacthit = numbacthit + 1;
            numcocktails = numcocktails + 1;
        }
    }

    for (i = 0; i < maxnumbershownField; i++) {
        if (numbacthit > cocktailhits[i][0]) {
            cocktailhits[i][0] = numbacthit;
            cocktailhits[i][1] = j0 + 1;
            cocktailhits[i][2] = j1 + 1;
            cocktailhits[i][3] = j2 + 1;
            cocktailhits[i][4] = j3 + 1;
            cocktailhits[i][5] = j4 + 1;
            cocktailhits[i][6] = j5 + 1;
            cocktailhits[i][7] = j6 + 1;
            cocktailhits[i][8] = j7 + 1;
            cocktailhits[i][9] = j8 + 1;
            cocktailhits[i][10] = j9 + 1;
            cocktailhits[i][11] = j10 + 1;
            cocktailhits[i][12] = j11 + 1;
            cocktailhits[i][13] = j12 + 1;
            cocktailhits[i][14] = j13 + 1;
            cocktailhits[i][15] = j14 + 1;
            i = maxnumbershownField;
        }
    }
    }

    }}}}}}}}}}}}}}}

//---------------------------------------------------//

if (maxsizecocktailField == 16) {
```

```
for (j0 = 0; j0 < numbersofphagesField-15; j0++) {
for (j1 = j0+1; j1 < numbersofphagesField-14; j1++) {
for (j2 = j1+1; j2 < numbersofphagesField-13; j2++) {
for (j3 = j2+1; j3 < numbersofphagesField-12; j3++) {
for (j4 = j3+1; j4 < numbersofphagesField-11; j4++) {
for (j5 = j4+1; j5 < numbersofphagesField-10; j5++) {
for (j6 = j5+1; j6 < numbersofphagesField-9; j6++) {
for (j7 = j6+1; j7 < numbersofphagesField-8; j7++) {
for (j8 = j7+1; j8 < numbersofphagesField-7; j8++) {
for (j9 = j8+1; j9 < numbersofphagesField-6; j9++) {
for (j10 = j9+1; j10 < numbersofphagesField-5; j10++) {
for (j11 = j10+1; j11 < numbersofphagesField-4; j11++) {
for (j12 = j11+1; j12 < numbersofphagesField-3; j12++) {
for (j13 = j12+1; j13 < numbersofphagesField-2; j13++) {
for (j14 = j13+1; j14 < numbersofphagesField-1; j14++) {
for (j15 = j14+1; j15 < numbersofphagesField; j15++) {

cocktailhits.sort(function(a, b) {
    return a[0] - b[0];
});

if (j15 > j14 && j14 > j13 && j13 > j12 && j12 > j11 && j11 > j10 && j10 > j9 && j9
> j8 && j8 > j7 && j7 > j6 && j6 > j5 && j5 > j4 && j4 > j3 && j3 > j2 && j2 > j1 && j1
> j0) {
numbacthit = 0;
for (k = 0; k < numbersofbacteriaField; k++) {

    if (TwoDArray[k][j0] + TwoDArray[k][j1] + TwoDArray[k][j2] + TwoDArray[k][j3] +
TwoDArray[k][j4] + TwoDArray[k][j5] + TwoDArray[k][j6] + TwoDArray[k][j7] + Two-
DArray[k][j8] + TwoDArray[k][j9] + TwoDArray[k][j10] + TwoDArray[k][j11] + Two-
DArray[k][j12] + TwoDArray[k][j13] + TwoDArray[k][j14] + TwoDArray[k][j15] > 0) {
        numbacthit = numbacthit + 1;
        numcocktails = numcocktails + 1;
    }
}

for (i = 0; i < maxnumbershownField; i++) {
    if (numbacthit > cocktailhits[i][0]) {
        cocktailhits[i][0] = numbacthit;
        cocktailhits[i][1] = j0 + 1;
        cocktailhits[i][2] = j1 + 1;
```

```
            cocktailhits[i][3] = j2 + 1;
            cocktailhits[i][4] = j3 + 1;
            cocktailhits[i][5] = j4 + 1;
            cocktailhits[i][6] = j5 + 1;
            cocktailhits[i][7] = j6 + 1;
            cocktailhits[i][8] = j7 + 1;
            cocktailhits[i][9] = j8 + 1;
            cocktailhits[i][10] = j9 + 1;
            cocktailhits[i][11] = j10 + 1;
            cocktailhits[i][12] = j11 + 1;
            cocktailhits[i][13] = j12 + 1;
            cocktailhits[i][14] = j13 + 1;
            cocktailhits[i][15] = j14 + 1;
            cocktailhits[i][16] = j15 + 1;
            i = maxnumbershownField;
        }
    }
    }

    }}}}}}}}}}}}}}}}}

//---------------------------------------------------//

if (maxsizecocktailField == 17) {

    for (j0 = 0; j0 < numbersofphagesField-16; j0++) {
    for (j1 = j0+1; j1 < numbersofphagesField-15; j1++) {
    for (j2 = j1+1; j2 < numbersofphagesField-14; j2++) {
    for (j3 = j2+1; j3 < numbersofphagesField-13; j3++) {
    for (j4 = j3+1; j4 < numbersofphagesField-12; j4++) {
    for (j5 = j4+1; j5 < numbersofphagesField-11; j5++) {
    for (j6 = j5+1; j6 < numbersofphagesField-10; j6++) {
    for (j7 = j6+1; j7 < numbersofphagesField-9; j7++) {
    for (j8 = j7+1; j8 < numbersofphagesField-8; j8++) {
    for (j9 = j8+1; j9 < numbersofphagesField-7; j9++) {
    for (j10 = j9+1; j10 < numbersofphagesField-6; j10++) {
    for (j11 = j10+1; j11 < numbersofphagesField-5; j11++) {
    for (j12 = j11+1; j12 < numbersofphagesField-4; j12++) {
    for (j13 = j12+1; j13 < numbersofphagesField-3; j13++) {
    for (j14 = j13+1; j14 < numbersofphagesField-2; j14++) {
    for (j15 = j14+1; j15 < numbersofphagesField-1; j15++) {
    for (j16 = j15+1; j16 < numbersofphagesField; j16++) {
```

```
    cocktailhits.sort(function(a, b) {
        return a[0] - b[0];
    });

    if (j16 > j15 && j15 > j14 && j14 > j13 && j13 > j12 && j12 > j11 && j11 > j10 &&
j10 > j9 && j9 > j8 && j8 > j7 && j7 > j6 && j6 > j5 && j5 > j4 && j4 > j3 && j3 > j2
&& j2 > j1 && j1 > j0) {
    numbacthit = 0;
    for (k = 0; k < numbersofbacteriaField; k++) {

        if (TwoDArray[k][j0] + TwoDArray[k][j1] + TwoDArray[k][j2] + TwoDArray[k][j3] +
TwoDArray[k][j4] + TwoDArray[k][j5] + TwoDArray[k][j6] + TwoDArray[k][j7] + Two-
DArray[k][j8] + TwoDArray[k][j9] + TwoDArray[k][j10] + TwoDArray[k][j11] + Two-
DArray[k][j12] + TwoDArray[k][j13] + TwoDArray[k][j14] + TwoDArray[k][j15] + Two-
DArray[k][j16] > numberofhitsField) {
            numbacthit = numbacthit + 1;
            numcocktails = numcocktails + 1;
        }
    }

    for (i = 0; i < maxnumbershownField; i++) {
        if (numbacthit > cocktailhits[i][0]) {
            cocktailhits[i][0] = numbacthit;
            cocktailhits[i][1] = j0 + 1;
            cocktailhits[i][2] = j1 + 1;
            cocktailhits[i][3] = j2 + 1;
            cocktailhits[i][4] = j3 + 1;
            cocktailhits[i][5] = j4 + 1;
            cocktailhits[i][6] = j5 + 1;
            cocktailhits[i][7] = j6 + 1;
            cocktailhits[i][8] = j7 + 1;
            cocktailhits[i][9] = j8 + 1;
            cocktailhits[i][10] = j9 + 1;
            cocktailhits[i][11] = j10 + 1;
            cocktailhits[i][12] = j11 + 1;
            cocktailhits[i][13] = j12 + 1;
            cocktailhits[i][14] = j13 + 1;
            cocktailhits[i][15] = j14 + 1;
            cocktailhits[i][16] = j15 + 1;
            cocktailhits[i][17] = j16 + 1;
            i = maxnumbershownField;
```

```
        }
    }
    }

    }}}}}}}}}}}}}}}}}

//---------------------------------------------------//

if (maxsizecocktailField == 18) {

    for (j0 = 0; j0 < numbersofphagesField-16; j0++) {
    for (j1 = j0+1; j1 < numbersofphagesField-15; j1++) {
    for (j2 = j1+1; j2 < numbersofphagesField-14; j2++) {
    for (j3 = j2+1; j3 < numbersofphagesField-13; j3++) {
    for (j4 = j3+1; j4 < numbersofphagesField-12; j4++) {
    for (j5 = j4+1; j5 < numbersofphagesField-11; j5++) {
    for (j6 = j5+1; j6 < numbersofphagesField-10; j6++) {
    for (j7 = j6+1; j7 < numbersofphagesField-9; j7++) {
    for (j8 = j7+1; j8 < numbersofphagesField-8; j8++) {
    for (j9 = j8+1; j9 < numbersofphagesField-7; j9++) {
    for (j10 = j9+1; j10 < numbersofphagesField-6; j10++) {
    for (j11 = j10+1; j11 < numbersofphagesField-5; j11++) {
    for (j12 = j11+1; j12 < numbersofphagesField-4; j12++) {
    for (j13 = j12+1; j13 < numbersofphagesField-3; j13++) {
    for (j14 = j13+1; j14 < numbersofphagesField-2; j14++) {
    for (j15 = j14+1; j15 < numbersofphagesField-1; j15++) {
    for (j16 = j15+1; j16 < numbersofphagesField; j16++) {
    for (j17 = j16+1; j17 < numbersofphagesField; j17++) {

    cocktailhits.sort(function(a, b) {
        return a[0] - b[0];
    });

    if (j17 > j16 && j16 > j15 && j15 > j14 && j14 > j13 && j13 > j12 && j12 > j11 &&
j11 > j10 && j10 > j9 && j9 > j8 && j8 > j7 && j7 > j6 && j6 > j5 && j5 > j4 && j4 > j3
&& j3 > j2 && j2 > j1 && j1 > j0) {
    numbacthit = 0;
    for (k = 0; k < numbersofbacteriaField; k++) {

        if (TwoDArray[k][j0] + TwoDArray[k][j1] + TwoDArray[k][j2] + TwoDArray[k][j3] +
TwoDArray[k][j4] + TwoDArray[k][j5] + TwoDArray[k][j6] + TwoDArray[k][j7] + Two-
DArray[k][j8] + TwoDArray[k][j9] + TwoDArray[k][j10] + TwoDArray[k][j11] +
```

```
TwoDArray[k][j12] + TwoDArray[k][j13] + TwoDArray[k][j14] + TwoDArray[k][j15] + Two-
DArray[k][j16] + TwoDArray[k][j17] > numberofhitsField) {
            numbacthit = numbacthit + 1;
            numcocktails = numcocktails + 1;
        }
    }

    for (i = 0; i < maxnumbershownField; i++) {
        if (numbacthit > cocktailhits[i][0]) {
            cocktailhits[i][0] = numbacthit;
            cocktailhits[i][1] = j0 + 1;
            cocktailhits[i][2] = j1 + 1;
            cocktailhits[i][3] = j2 + 1;
            cocktailhits[i][4] = j3 + 1;
            cocktailhits[i][5] = j4 + 1;
            cocktailhits[i][6] = j5 + 1;
            cocktailhits[i][7] = j6 + 1;
            cocktailhits[i][8] = j7 + 1;
            cocktailhits[i][9] = j8 + 1;
            cocktailhits[i][10] = j9 + 1;
            cocktailhits[i][11] = j10 + 1;
            cocktailhits[i][12] = j11 + 1;
            cocktailhits[i][13] = j12 + 1;
            cocktailhits[i][14] = j13 + 1;
            cocktailhits[i][15] = j14 + 1;
            cocktailhits[i][16] = j15 + 1;
            cocktailhits[i][17] = j16 + 1;
            cocktailhits[i][18] = j17 + 1;
            i = maxnumbershownField;
        }
    }
    }

    }}}}}}}}}}}}}}}}}}

//---------------------------------------------------//

if (maxsizecocktailField == 19) {

    for (j0 = 0; j0 < numbersofphagesField-16; j0++) {
    for (j1 = j0+1; j1 < numbersofphagesField-15; j1++) {
    for (j2 = j1+1; j2 < numbersofphagesField-14; j2++) {
```

```
    for (j3 = j2+1; j3 < numbersofphagesField-13; j3++) {
    for (j4 = j3+1; j4 < numbersofphagesField-12; j4++) {
    for (j5 = j4+1; j5 < numbersofphagesField-11; j5++) {
    for (j6 = j5+1; j6 < numbersofphagesField-10; j6++) {
    for (j7 = j6+1; j7 < numbersofphagesField-9; j7++) {
    for (j8 = j7+1; j8 < numbersofphagesField-8; j8++) {
    for (j9 = j8+1; j9 < numbersofphagesField-7; j9++) {
    for (j10 = j9+1; j10 < numbersofphagesField-6; j10++) {
    for (j11 = j10+1; j11 < numbersofphagesField-5; j11++) {
    for (j12 = j11+1; j12 < numbersofphagesField-4; j12++) {
    for (j13 = j12+1; j13 < numbersofphagesField-3; j13++) {
    for (j14 = j13+1; j14 < numbersofphagesField-2; j14++) {
    for (j15 = j14+1; j15 < numbersofphagesField-1; j15++) {
    for (j16 = j15+1; j16 < numbersofphagesField; j16++) {
    for (j17 = j16+1; j17 < numbersofphagesField; j17++) {
    for (j18 = j17+1; j18 < numbersofphagesField; j18++) {

    cocktailhits.sort(function(a, b) {
        return a[0] - b[0];
    });

    if (j18 > j17 && j17 > j16 && j16 > j15 && j15 > j14 && j14 > j13 && j13 > j12 &&
j12 > j11 && j11 > j10 && j10 > j9 && j9 > j8 && j8 > j7 && j7 > j6 && j6 > j5 && j5 >
j4 && j4 > j3 && j3 > j2 && j2 > j1 && j1 > j0) {
    numbacthit = 0;
    for (k = 0; k < numbersofbacteriaField; k++) {

        if (TwoDArray[k][j0] + TwoDArray[k][j1] + TwoDArray[k][j2] + TwoDArray[k][j3] +
TwoDArray[k][j4] + TwoDArray[k][j5] + TwoDArray[k][j6] + TwoDArray[k][j7] + Two-
DArray[k][j8] + TwoDArray[k][j9] + TwoDArray[k][j10] + TwoDArray[k][j11] + Two-
DArray[k][j12] + TwoDArray[k][j13] + TwoDArray[k][j14] + TwoDArray[k][j15] + Two-
DArray[k][j16] + TwoDArray[k][j17] + TwoDArray[k][j18] > numberofhitsField) {
            numbacthit = numbacthit + 1;
            numcocktails = numcocktails + 1;
        }
    }

    for (i = 0; i < maxnumbershownField; i++) {
        if (numbacthit > cocktailhits[i][0]) {
            cocktailhits[i][0] = numbacthit;
            cocktailhits[i][1] = j0 + 1;
            cocktailhits[i][2] = j1 + 1;
```

```
            cocktailhits[i][3] = j2 + 1;
            cocktailhits[i][4] = j3 + 1;
            cocktailhits[i][5] = j4 + 1;
            cocktailhits[i][6] = j5 + 1;
            cocktailhits[i][7] = j6 + 1;
            cocktailhits[i][8] = j7 + 1;
            cocktailhits[i][9] = j8 + 1;
            cocktailhits[i][10] = j9 + 1;
            cocktailhits[i][11] = j10 + 1;
            cocktailhits[i][12] = j11 + 1;
            cocktailhits[i][13] = j12 + 1;
            cocktailhits[i][14] = j13 + 1;
            cocktailhits[i][15] = j14 + 1;
            cocktailhits[i][16] = j15 + 1;
            cocktailhits[i][17] = j16 + 1;
            cocktailhits[i][18] = j17 + 1;
            cocktailhits[i][19] = j18 + 1;
            i = maxnumbershownField;
        }
    }
    }

    }}}}}}}}}}}}}}}}}}}

//---------------------------------------------------//

if (maxsizecocktailField == 20) {

    for (j0 = 0; j0 < numbersofphagesField-16; j0++) {
    for (j1 = j0+1; j1 < numbersofphagesField-15; j1++) {
    for (j2 = j1+1; j2 < numbersofphagesField-14; j2++) {
    for (j3 = j2+1; j3 < numbersofphagesField-13; j3++) {
    for (j4 = j3+1; j4 < numbersofphagesField-12; j4++) {
    for (j5 = j4+1; j5 < numbersofphagesField-11; j5++) {
    for (j6 = j5+1; j6 < numbersofphagesField-10; j6++) {
    for (j7 = j6+1; j7 < numbersofphagesField-9; j7++) {
    for (j8 = j7+1; j8 < numbersofphagesField-8; j8++) {
    for (j9 = j8+1; j9 < numbersofphagesField-7; j9++) {
    for (j10 = j9+1; j10 < numbersofphagesField-6; j10++) {
    for (j11 = j10+1; j11 < numbersofphagesField-5; j11++) {
    for (j12 = j11+1; j12 < numbersofphagesField-4; j12++) {
    for (j13 = j12+1; j13 < numbersofphagesField-3; j13++) {
```

```
    for (j14 = j13+1; j14 < numbersofphagesField-2; j14++) {
    for (j15 = j14+1; j15 < numbersofphagesField-1; j15++) {
    for (j16 = j15+1; j16 < numbersofphagesField; j16++) {
    for (j17 = j16+1; j17 < numbersofphagesField; j17++) {
    for (j18 = j17+1; j18 < numbersofphagesField; j18++) {
    for (j19 = j18+1; j19 < numbersofphagesField; j19++) {

    cocktailhits.sort(function(a, b) {
        return a[0] - b[0];
    });

    if (j19 > j18 && j18 > j17 && j17 > j16 && j16 > j15 && j15 > j14 && j14 > j13 &&
j13 > j12 && j12 > j11 && j11 > j10 && j10 > j9 && j9 > j8 && j8 > j7 && j7 > j6 && j6
> j5 && j5 > j4 && j4 > j3 && j3 > j2 && j2 > j1 && j1 > j0) {
    numbacthit = 0;
    for (k = 0; k < numbersofbacteriaField; k++) {

        if (TwoDArray[k][j0] + TwoDArray[k][j1] + TwoDArray[k][j2] + TwoDArray[k][j3] +
TwoDArray[k][j4] + TwoDArray[k][j5] + TwoDArray[k][j6] + TwoDArray[k][j7] + Two-
DArray[k][j8] + TwoDArray[k][j9] + TwoDArray[k][j10] + TwoDArray[k][j11] + Two-
DArray[k][j12] + TwoDArray[k][j13] + TwoDArray[k][j14] + TwoDArray[k][j15] + Two-
DArray[k][j16] + TwoDArray[k][j17] + TwoDArray[k][j18] + TwoDArray[k][j19] > numberof-
hitsField) {
            numbacthit = numbacthit + 1;
            numcocktails = numcocktails + 1;
        }
    }

    for (i = 0; i < maxnumbershownField; i++) {
        if (numbacthit > cocktailhits[i][0]) {
            cocktailhits[i][0] = numbacthit;
            cocktailhits[i][1] = j0 + 1;
            cocktailhits[i][2] = j1 + 1;
            cocktailhits[i][3] = j2 + 1;
            cocktailhits[i][4] = j3 + 1;
            cocktailhits[i][5] = j4 + 1;
            cocktailhits[i][6] = j5 + 1;
            cocktailhits[i][7] = j6 + 1;
            cocktailhits[i][8] = j7 + 1;
            cocktailhits[i][9] = j8 + 1;
            cocktailhits[i][10] = j9 + 1;
            cocktailhits[i][11] = j10 + 1;
```

```
            cocktailhits[i][12] = j11 + 1;
            cocktailhits[i][13] = j12 + 1;
            cocktailhits[i][14] = j13 + 1;
            cocktailhits[i][15] = j14 + 1;
            cocktailhits[i][16] = j15 + 1;
            cocktailhits[i][17] = j16 + 1;
            cocktailhits[i][18] = j17 + 1;
            cocktailhits[i][19] = j18 + 1;
            cocktailhits[i][20] = j19 + 1;
            i = maxnumbershownField;
        }
    }
    }

    }}}}}}}}}}}}}}}}}}}}}

//---------------------------------------------------//

cocktailhits.sort(function(a, b) {
    return b[0] - a[0];
});

}

//////////////////////////////////////////////////////////////////////////////
// OUTPUT

    if (errorvar == 'F') {

    result1.textContent = 'numbers of phages tested = ' + numbersofphagesField;
    result2.textContent = 'numbers of bacteria tested = ' + numbersofbacteriaField + '
(# of rows = ' + k + ')';
    result3.textContent = 'number of phages per optimized cocktail = ' + maxsizecock-
tailField;
    result4.textContent = 'number phages impacting each \'hit\' bacterium = ' + (num-
berofhitsField+1);
    result5.textContent = 'number optimized cocktails shown = ' + maxnumbershownField;
    result6.textContent = 'number of phage combinations tested = ' + numcocktails;
    result7.textContent = 'below, \'# hits\' = # of bacteria targeted by > ' + (number-
ofhitsField);
    if (numberofhitsField == 1) {
    result7.textContent = result7.textContent + ' phage';
```

```
    }
    else
    {
    result7.textContent = result7.textContent + ' phages';
    }

    var outputstr = new Array(maxnumbershownField);
    for (i = 0; i < maxnumbershownField; i++) {
        outputstr[i] = '';
    }


    var spacestr = ',';
    if (maxsizecocktailField < 7) {
            spacestr = ', ';
        }

    var frontstr = 'cocktail #';
    if (maxsizecocktailField > 8) {
            frontstr = '#';
    }

    var hitsstr = '';
    if (maxsizecocktailField < 10) {
            hitsstr = ' = # hits';
    }

    for (i = 0; i < maxnumbershownField; i++) {

        if (maxsizecocktailField > 1 && cocktailhits[i][1] != 0) {
             outputstr[i] = frontstr + (i+1) + ', ' + (Math.round(10000*cock-
tailhits[i][0]/numbersofbacteriaField)/100) + '%, ' + cocktailhits[i][0] + hitsstr + ':
' + cocktailhits[i][1] + spacestr + cocktailhits[i][2];
        }
        if (maxsizecocktailField > 2 && cocktailhits[i][1] != 0) { outputstr[i] = out-
putstr[i] +  spacestr + cocktailhits[i][3]; }
        if (maxsizecocktailField > 3 && cocktailhits[i][1] != 0) { outputstr[i] = out-
putstr[i] +  spacestr + cocktailhits[i][4]; }
        if (maxsizecocktailField > 4 && cocktailhits[i][1] != 0) { outputstr[i] = out-
putstr[i] +  spacestr + cocktailhits[i][5]; }
        if (maxsizecocktailField > 5 && cocktailhits[i][1] != 0) { outputstr[i] = out-
putstr[i] +  spacestr + cocktailhits[i][6]; }
```

```
        if (maxsizecocktailField > 6 && cocktailhits[i][1] != 0) { outputstr[i] = out-
putstr[i] +  spacestr + cocktailhits[i][7]; }
        if (maxsizecocktailField > 7 && cocktailhits[i][1] != 0) { outputstr[i] = out-
putstr[i] +  spacestr + cocktailhits[i][8]; }
        if (maxsizecocktailField > 8 && cocktailhits[i][1] != 0) { outputstr[i] = out-
putstr[i] +  spacestr + cocktailhits[i][9]; }
        if (maxsizecocktailField > 9 && cocktailhits[i][1] != 0) { outputstr[i] = out-
putstr[i] +  spacestr + cocktailhits[i][10]; }
        if (maxsizecocktailField > 10 && cocktailhits[i][1] != 0) { outputstr[i] = out-
putstr[i] +  spacestr + cocktailhits[i][11]; }
        if (maxsizecocktailField > 11 && cocktailhits[i][1] != 0) { outputstr[i] = out-
putstr[i] +  spacestr + cocktailhits[i][12]; }
        if (maxsizecocktailField > 12 && cocktailhits[i][1] != 0) { outputstr[i] = out-
putstr[i] +  spacestr + cocktailhits[i][13]; }
        if (maxsizecocktailField > 13 && cocktailhits[i][1] != 0) { outputstr[i] = out-
putstr[i] +  spacestr + cocktailhits[i][14]; }
        if (maxsizecocktailField > 14 && cocktailhits[i][1] != 0) { outputstr[i] = out-
putstr[i] +  spacestr + cocktailhits[i][15]; }
        if (maxsizecocktailField > 15 && cocktailhits[i][1] != 0) { outputstr[i] = out-
putstr[i] +  spacestr + cocktailhits[i][16]; }
        if (maxsizecocktailField > 16 && cocktailhits[i][1] != 0) { outputstr[i] = out-
putstr[i] +  spacestr + cocktailhits[i][17]; }
        if (maxsizecocktailField > 17 && cocktailhits[i][1] != 0) { outputstr[i] = out-
putstr[i] +  spacestr + cocktailhits[i][18]; }
        if (maxsizecocktailField > 18 && cocktailhits[i][1] != 0) { outputstr[i] = out-
putstr[i] +  spacestr + cocktailhits[i][19]; }
        if (maxsizecocktailField > 19 && cocktailhits[i][1] != 0) { outputstr[i] = out-
putstr[i] +  spacestr + cocktailhits[i][20]; }
        if (maxsizecocktailField > 20 && cocktailhits[i][1] != 0) { outputstr[i] = out-
putstr[i] +  spacestr + cocktailhits[i][21]; }

    }

    result8.textContent = outputstr[0];
    result9.textContent = outputstr[1];
    result10.textContent = outputstr[2];
    result12.textContent = outputstr[3];
    result13.textContent = outputstr[4];
    result14.textContent = outputstr[5];
    result15.textContent = outputstr[6];
    result16.textContent = outputstr[7];
    result17.textContent = outputstr[8];
```

```
result18.textContent = outputstr[9];
result19.textContent = outputstr[10];
result20.textContent = outputstr[11];
result21.textContent = outputstr[12];
result22.textContent = outputstr[13];
result23.textContent = outputstr[14];
result24.textContent = outputstr[15];
result25.textContent = outputstr[16];
result26.textContent = outputstr[17];
result27.textContent = outputstr[18];
result28.textContent = outputstr[19];
result29.textContent = outputstr[20];
result30.textContent = outputstr[21];
result31.textContent = outputstr[22];
result32.textContent = outputstr[23];
result33.textContent = outputstr[24];
result34.textContent = outputstr[25];
result35.textContent = outputstr[26];
result36.textContent = outputstr[27];
result37.textContent = outputstr[28];
result38.textContent = outputstr[29];
result39.textContent = outputstr[30];
result40.textContent = outputstr[31];
result41.textContent = outputstr[32];
result42.textContent = outputstr[33];
result43.textContent = outputstr[34];
result44.textContent = outputstr[35];
result45.textContent = outputstr[36];
result46.textContent = outputstr[37];
result47.textContent = outputstr[38];
result48.textContent = outputstr[39];
result49.textContent = outputstr[40];
result50.textContent = outputstr[41];
result51.textContent = outputstr[42];
result52.textContent = outputstr[43];
result53.textContent = outputstr[44];
result54.textContent = outputstr[45];
result55.textContent = outputstr[46];
result56.textContent = outputstr[47];
result57.textContent = outputstr[48];
result58.textContent = outputstr[49];
result59.textContent = outputstr[50];
```

```
    result60.textContent = '----------------------------------------------------------
----------';

    var end = new Date();
    var elapsed_time = (end.getTime() - start.getTime());
    var timestr = elapsed_time + ' milliseconds';
    if (elapsed_time = 1) {
        timestr = '1 millisecond';
    }
    if (elapsed_time = 1000) {
        timestr = '1 second';
    }
    if (elapsed_time > 1000) {
        elapsed_time = elapsed_time/1000;
        timestr = elapsed_time.toFixed(1) + ' seconds';
        if (elapsed_time.toFixed(1) = 1) {
        timestr = '1 second';
        }
    }
    result61.textContent = 'total elapsed run time = ' + timestr;
   }

    else {

    result3.textContent = ' ';
    result4.textContent = ' ';
    result5.textContent = ' ';
    result6.textContent = ' ';
    result7.textContent = ' ';
    result8.textContent = ' ';
    result9.textContent = ' ';
    result10.textContent = ' ';
    result11.textContent = ' ';
    result12.textContent = ' ';
    result13.textContent = ' ';
    result14.textContent = ' ';
    result15.textContent = ' ';
    result16.textContent = ' ';
    result17.textContent = ' ';
    result18.textContent = ' ';
    result19.textContent = ' ';
    result20.textContent = ' ';
```

```
result21.textContent = ' ';
result22.textContent = ' ';
result23.textContent = ' ';
result24.textContent = ' ';
result25.textContent = ' ';
result26.textContent = ' ';
result27.textContent = ' ';
result28.textContent = ' ';
result29.textContent = ' ';
result30.textContent = ' ';
result31.textContent = ' ';
result32.textContent = ' ';
result33.textContent = ' ';
result34.textContent = ' ';
result35.textContent = ' ';
result36.textContent = ' ';
result37.textContent = ' ';
result38.textContent = ' ';
result39.textContent = ' ';
result40.textContent = ' ';
result41.textContent = ' ';
result42.textContent = ' ';
result43.textContent = ' ';
result44.textContent = ' ';
result45.textContent = ' ';
result46.textContent = ' ';
result47.textContent = ' ';
result48.textContent = ' ';
result49.textContent = ' ';
result50.textContent = ' ';
result51.textContent = ' ';
result52.textContent = ' ';
result53.textContent = ' ';
result54.textContent = ' ';
result55.textContent = ' ';
result56.textContent = ' ';
result57.textContent = ' ';
result58.textContent = ' ';
result59.textContent = ' ';
result60.textContent = ' ';
result61.textContent = ' ';
```

```
    }

}

// use an eventlistener for the event
var subButton1 = document.getElementById('subButton1');
subButton1.addEventListener('click',cocktail_optimizer, false);
```

S4. www.phage-therapy.org/calculators/xresistance_avoider.html [return to Table of Contents]

```
<!DOCTYPE html PUBLIC "-//W3C//DTD XHTML 1.0 Transitional//EN" "http://www.w3.org/TR/xhtml1/DTD/xhtml1-transitional.dtd">
<html xmlns="http://www.w3.org/1999/xhtml">
<head><meta charset="iso-8859-1">

<title>Cross-Resistance Avoider - Bacteriophage Ecology Group</title>

<meta property="og:image" content="/images/logo.png"/>

<META name="description" content= "Phage Cocktail Cross-Resistance Avoider | How well will your phage cocktail impact your favorite bacterial species in light of cross resistance? |  " />

<META name="keywords" content=

Stephen T. Abedon,
Stephen Abedon,
Steve Abedon,
S. T. Abedon,
S.T. Abedon,
S. Abedon,
ST Abedon,
S Abedon,
SAbedon,
Abedon,
Cocktail,
Phage Cocktail,
Phage Cocktail Avoider,
Phage Cocktail Cross Resistance Avoider,
Cross Resistance,
Resistance,
Phage Resistance,
Spot Test,
Spot Assay,
Host Range,
Maximize Host Range,
Phage Host Range,
Spectrum of Activity,
Cocktail Spectrum of Activity,
Cocktail Host Range

>
```

```
<link href="http://www.BiologyAsPoetry.com/css/terms_import_BaP.css" rel="stylesheet" type="text/css" />
<link href="http://www.BiologyAsPoetry.com/css/forms.css" rel="stylesheet" type="text/css" />
<link rel="icon" href="/favicon.ico" />

<script type="text/javascript" src="http://www.BiologyAsPoetry.com/scripts/other_links.js"></script>
<script type="text/javascript" src="http://www.BiologyAsPoetry.com/scripts/front_material.js"> </script>

<script type="text/javascript">

  var _gaq = _gaq || [];
  _gaq.push(['_setAccount', 'UA-23749643-1']);
  _gaq.push(['_trackPageview']);

  (function() {
    var ga = document.createElement('script'); ga.type = 'text/javascript'; ga.async = true;
    ga.src = ('https:' == document.location.protocol ? 'https://ssl' : 'http://www') + '.google-analyt-
ics.com/ga.js';
    var s = document.getElementsByTagName('script')[0]; s.parentNode.insertBefore(ga, s);
  })();

</script>

</head>

<body>

<div id="fb-root"></div>
<script>(function(d, s, id) {
  var js, fjs = d.getElementsByTagName(s)[0];
  if (d.getElementById(id)) return;
  js = d.createElement(s); js.id = id;
  js.src = "//connect.facebook.net/en_US/sdk.js#xfbml=1&version=v2.0";
  fjs.parentNode.insertBefore(js, fjs);
}(document, 'script', 'facebook-jssdk'));</script>

<script type="text/javascript" src="http://www.google.com/jsapi"></script>

<script type="text/javascript"> IsPrivate(); </script> <script type="text/javascript"> front_mate-
rial("phage_therapy","Phage Cocktail<BR>Cross-Resistance Avoider","calculate your complex cocktail's depth
and breadth of activity","F","F","F"); </script>
```

```
<H1 id="TitleTransparent"><font color=transparent>Phage Cocktail Cross-Resistance Avoider</font></H1>

</P><P id="DefinitionCenter"><font color=#A0A0A0 size=2>&infin; generated and posted on
2020.10.22 &infin;</font></P>

</P><P id="DefinitionCenterEmphasis">How well will your phage cocktail impact your favorite bacterial species
in light of cross resistance?

<script type="text/javascript"> other_links("TX","Cross Resistnace Avoider","F","F","F","F","BEG","F");
</script>

<! ///////////////////////////////////////////////////////////////////////////////////////>

<! Place Dropdown box here >

<BR><BR>

<form name="nav">
<select id="listbox_positioning_phagetherapyorg" name="SelectURL" onchange="document.location.href=docu-
ment.nav.SelectURL.options[document.nav.SelectURL.selectedIndex].value">
  <option value="index.html">Quick Navigation of Phage-Therapy.org</option>
  <option value="http://phage-therapy.org/writings/bacteriophages.html">What are Phages as well as Phage
Therapy?</option>
  <option value="http://publications.phage-therapy.org">Phage Therapy Publications</option>
  <option value="videos.html">Phage Therapy Videos</option>
  <option value="advantages.html">Advantages of Phage Therapy</option>
  <option value="http://www.phage-therapy.org/virtual_book.html">Phage Therapy Virtual "Book"</option>
  <option value="http://companies.phage.org">Phage Companies</option>Killing Titer Calculator
  <option value="http://www.phage.org">Phage.org (Bacteriophage Ecology Group)</option>
  <option value="http://www.phage-therapy.org/resources.html">Summary of Content/Pages/Links</option>
  <option value="http://www.phage-therapy.org">Phage-Therapy.org Main Page</option>
</select>
</form>

<! ///////////////////////////////////////////////////////////////////////////////////////>


</P><P id="DefinitionCenter"><font color=red>Please cite as:</font><BR><BR><font color=#8A5C00><B>Stephen T.
Abedon</B><BR>Phage Cocktail Cross-Resistance Avoider<BR><a onclick="parent.location = 'http://www.phage-
therapy.org/calculators/xresistance_avoider.html'"><u>phage-therapy.org/calculators/xre-
sistance_avoider.html</u></a></font><BR><BR>
```

```
</P><div id="poetry_definition_positioning"><table id="poetry_definition" cellspacing="2"><tr><td class="po-
etry_definition_text"><font color=red>This page builds on that of <a href="http://www.phage-therapy.org/cal-
culators/cocktail_optimizer.html" title="Phage Cocktail Optimizer">http://www.phage-therapy.org/calcula-
tors/cocktail_optimizer.html</a> by taking into account the cross-resistance groups that individual phages in
a cocktail are found in. It does this by determining the degrees of host range-overlap for every combination
of phages found within a cocktail that are not found in the same cross-resitance group.<BR><BR>For example,
for three cross-resistance groups &ndash; 1, 2, and 3 &ndash; there may be 0 phages (e.g., as found in 00000,
00000, and 00000), 1 phage (e.g., 00000, 01010, and 00000), 2 phages (e.g., 00001, 01010, and 00000), or 3
phages (e.g., 00001, 01010, and 01000) that are found in different cross-resistance groups (the different
groups of 1s and 0s), representing depths of activity against the single bacterium in question of 0, 1, 2,
and 3, respectively.<BR><BR><font color=gray>Initially, as written, this analysis will be limited to as-
sessing no more than two cross resistance groups.</font><BR><BR>To run this app, your goal will be to gener-
ate two or more two-dimensional arrays consisting of a stack of one-dimensional arrays (i.e., rows) with each
row corresponding to one potential target baterial strain that has been tested for phage host range. Arrays
should consist of ones and zeros only, with each digit, whether positive (1) or negative (0), referring to a
single tested phage type.<BR><BR>An example could be "10110", which would represent one tested bacterial
strain and 5 different tested phages, for three of which that bacterium is found within their host range
(corresponding to the three 1s). The length of this array/row thus will be that of the number of phages
tested, i.e., in the example this would be 5 phages.<BR><BR>The number of rows will be the number bacteria
tested and order and number of rows should be kept constant from box to box (and should equal the "Number of
bacteria tested" as indicated below). Thus, rows represent the data for a single bacterium and columns repre-
sent the data for a single phage, with explicitely different phages found in different boxes (these boxes are
found within the 'blue', below).<BR><BR>For output, note that rows are presented as a list showing calculated
phage depth of activity for each bacterium tested, with each digit corresponding, in order, to each row. For
example, "2,1,0,1,2,0,3,1" would indicate that one bacterium is hit by three phages from three different
cross-reistance groups, two bacteria are hit by two phages from two different cross-resistance groups, three
bacteria are hit by only one phage each, and two bacteria are hit by no phages among those
tested.<BR><BR><B>Only bacteria hit by at least two phages that are members of at least two different cross-
resistance groups (depth of activity > 1) will display a reduced potential to mutationally evolve phage re-
sistance.</B><BR><BR>Each box (see as found within the 'blue' below) should correspond to the host-range data
for phages found within a single cross-resistance group.<BR><BR>Here is some test phage host-range data to
try, separated into three different cross-resistance groups:<BR><BR>

    <table style="margin-left: auto; margin-right: auto;"><tr><td>
    <table>

    <tr><th>  Test xRes  <BR>  Group #1:  <BR><BR></th></tr>

    <tr><td><font face="Courier New" size=5 color=black>00000</td></tr>
    <tr><td><font face="Courier New" size=5 color=black>10110</td></tr>
    <tr><td><font face="Courier New" size=5 color=black>01000</td></tr>
    <tr><td><font face="Courier New" size=5 color=black>00000</td></tr>
```

```
<tr><td><font face="Courier New" size=5 color=black>00000</td></tr>
<tr><td><font face="Courier New" size=5 color=black>00000</td></tr>
<tr><td><font face="Courier New" size=5 color=black>01000</td></tr>
<tr><td><font face="Courier New" size=5 color=black>01010</td></tr>
<tr><td><font face="Courier New" size=5 color=black>11100</td></tr>
<tr><td><font face="Courier New" size=5 color=black>01100</td></tr>

</table>
</td>
<td>
<table>
<tr><th>  Test xRes  <BR>  Group #2:  <BR><BR></th></tr>

<tr><td><font face="Courier New" size=5 color=black>10110</td></tr>
<tr><td><font face="Courier New" size=5 color=black>01010</td></tr>
<tr><td><font face="Courier New" size=5 color=black>11000</td></tr>
<tr><td><font face="Courier New" size=5 color=black>00000</td></tr>
<tr><td><font face="Courier New" size=5 color=black>00101</td></tr>
<tr><td><font face="Courier New" size=5 color=black>00000</td></tr>
<tr><td><font face="Courier New" size=5 color=black>00011</td></tr>
<tr><td><font face="Courier New" size=5 color=black>11010</td></tr>
<tr><td><font face="Courier New" size=5 color=black>00000</td></tr>
<tr><td><font face="Courier New" size=5 color=black>01000</td></tr>

</table>
</td>
<td>
<table>
<tr><th>  Test xRes  <BR>  Group #3:  <BR><BR></th></tr>

<tr><td><font face="Courier New" size=5 color=black>00000</td></tr>
<tr><td><font face="Courier New" size=5 color=black>10110</td></tr>
<tr><td><font face="Courier New" size=5 color=black>00000</td></tr>
<tr><td><font face="Courier New" size=5 color=black>00000</td></tr>
<tr><td><font face="Courier New" size=5 color=black>11100</td></tr>
<tr><td><font face="Courier New" size=5 color=black>00000</td></tr>
<tr><td><font face="Courier New" size=5 color=black>01011</td></tr>
<tr><td><font face="Courier New" size=5 color=black>00000</td></tr>
<tr><td><font face="Courier New" size=5 color=black>00001</td></tr>
<tr><td><font face="Courier New" size=5 color=black>00000</td></tr>

</table>
```

```
    </td>
    <td>
    <table>
    <tr><th>  Test xRes  <BR>  Group #4:  <BR><BR></th></tr>

    <tr><td><font face="Courier New" size=5 color=black>00000</td></tr>
    <tr><td><font face="Courier New" size=5 color=black>01100</td></tr>
    <tr><td><font face="Courier New" size=5 color=black>00000</td></tr>
    <tr><td><font face="Courier New" size=5 color=black>00010</td></tr>
    <tr><td><font face="Courier New" size=5 color=black>00000</td></tr>
    <tr><td><font face="Courier New" size=5 color=black>00000</td></tr>
    <tr><td><font face="Courier New" size=5 color=black>00111</td></tr>
    <tr><td><font face="Courier New" size=5 color=black>00000</td></tr>
    <tr><td><font face="Courier New" size=5 color=black>01100</td></tr>
    <tr><td><font face="Courier New" size=5 color=black>00100</td></tr>

    </table> </td></tr></table>

    <BR>

    </td></tr></table></div>

<a name="calculator">

</P><div id="facebookpositionmainpagecenter"><div class="fb-like" data-href="http://Phage-Therapy.org/calcu-
lators/xresistance_avoider.html" data-layout="standard" data-action="like" data-show-faces="true" data-
share="true"></div></div>

</a>

</P><P id="Definition"><!BR><!BR></P>

  <div class='wrapper'>
    <BR>
    <form id='nameForm'>
    <div class='form-uname'>
        <label><font color=blue><B>Optimize Cocktail Spectrum of Activity</B></label><BR><BR>

        <label id='numbersof' for='numbersofField'><B>Enter the number of phages and hosts tested:</B></la-
bel>

        <BR><BR>
```

```
<label id='numbersofphages0' for='numbersofphagesField0'>Number of phages tested:<BR><BR></label>
<label id='numbersofphages1' for='numbersofphagesField1'>Cross Resistance Group #1 =  </label>
<input id='numbersofphagesField1' type='number' maxlength='25' value="5"></input>
<label id='numbersofphages2' for='numbersofphagesField2'>Cross Resistance Group #2 =  </label>
<input id='numbersofphagesField2' type='number' maxlength='25' value="5"></input>
<label id='numbersofphages3' for='numbersofphagesField3'>Cross Resistance Group #3 =  </label>
<input id='numbersofphagesField3' type='number' maxlength='25' value="5"></input>
<label id='numbersofphages4' for='numbersofphagesField4'>Cross Resistance Group #4 =  </label>
<input id='numbersofphagesField4' type='number' maxlength='25' value="5"></input>
<BR><font size=3 color=red>Above is the <I>number</I> of 1s and 0s (phages) per row.</font>

<BR><BR>

<label id='numbersofbacteria' for='numbersofbacteriaField'>   Number of bacteria
tested =  </label>
<input id='numbersofbacteriaField' type='number' maxlength='50' value="10"></input>
<BR><font size=3 color=red>Above is the <I>number</I> of rows (bacteria) of 1s and 0s.</font>

<BR><BR>

<label id='TwoDarrays' for='TwoDarrayField0'><B>Enter 2D Arrays of Host Range Results:</B><BR></la-
bel>

<label id='Instructions' for='Instructions'>1=pos pheno, rows=hosts, columns (digits)=phages<BR><font
size=3 color=red>Boxes refer to different phage cross-resitance groups.</font><BR><BR><font size=3>xRes group
#1: xRes group #2: xRes group #3: xRes group #4:<BR></label>

<textarea id="TwoDarrayField1" name="subject" placeholder="010101100001 100100011000 001011000010
000100110111 000000000000 000000011001 001010000010 000001000111 000000100000 110100000010 000000010001
000100000010 000000001000 100110110010 000000010011" rows="15" cols="11"></textarea>

<textarea id="TwoDarrayField2" name="subject" placeholder="110100000000 000000010001 000100000011
010101100001 100100011010 001011000010 000100110100 000000000000 000000011011 001010000001 000001000101
000000100011 000000001010 100110110000 000000010011" rows="15" cols="11"></textarea>

<textarea id="TwoDarrayField3" name="subject" placeholder="000000100001 110100000001 000000010010
000100000000 000000001001 100110110011 000000010010 010101100011 100100011010 001011000011 000100110101
000000000011 000000011000 001010000001 000001000111" rows="15" cols="11"></textarea>

<textarea id="TwoDarrayField4" name="subject" placeholder="000001000110 000000100011 110100000001
000000010000 000100000000 000000001001 100110110010 000000010011 001011000001 010101100011 100100011001
000100110101 000000000001 000000011000 001010000010" rows="15" cols="11"></textarea>
```

```
        <BR><!BR>

        <BR><font size=3 color=red>1 means pos result, e.g., non-turbid spot (some colonies OK).</font>
        <BR><font size=3 color=red>0 means neg result, e.g., turbid spot or individual plaques.</font>
        <BR><font size=3 color=red>010 means first phage is neg, second is pos, third is neg&hellip;</font>
        <BR><font size=3 color=red>Start a new row to enter data from an additional host.</font>
        <BR><font size=3 color=red>Make sure that each row is separated by a break line.</font>
        <BR><font size=3 color=red>Note that xRes Group #3 need not be entered.</font>

     </div>
 <BR>
   <div class='form-sub'>
        <button id='subButton1' type='button'>Optimize Cocktail Spectrum of Activity</button>
        <button type="reset" value="Reset">Reset Form</button>
     </div>
</form>

<! Code is based on that found here: http://codepen.io/NickersF/pen/EVjVNd >

<div>
    <p id='result0'></p></div>
<div>
    <p id='result1'></p></div>
<div>
    <p id='result2'></p></div>
<div>
    <p id='result3'></p></div>
<div>
    <p id='result4'></p></div>
<div>
    <p id='result5'></p></div>
<div>
    <p id='result6'></p></div>
<div>
    <p id='result7'></p></div>
<div>
    <p id='result8'></p></div>
<div>
    <p id='result9'></p></div>
<div>
    <p id='result10'></p></div>
```

```
<div>
    <p id='result11'></p></div>
<div>
    <p id='result12'></p></div>
<div>
    <p id='result13'></p></div>
<div>
    <p id='result14'></p></div>
<div>
    <p id='result15'></p></div>
<div>
    <p id='result16'></p></div>
<div>
    <p id='result17'></p></div>
<div>
    <p id='result18'></p></div>
<div>
    <p id='result19'></p></div>
<div>
    <p id='result20'></p></div>
<div>
    <p id='result21'></p></div>
<div>
    <p id='result22'></p></div>
<div>
    <p id='result23'></p></div>
<div>
    <p id='result24'></p></div>
<div>
    <p id='result25'></p></div>
<div>
    <p id='result26'></p></div>
<div>
    <p id='result27'></p></div>
<div>
    <p id='result28'></p></div>
<div>
    <p id='result29'></p></div>
<div>
    <p id='result30'></p></div>
<div>
    <p id='result31'></p></div>
```

```
<div>
    <p id='result32'></p></div>
<div>
    <p id='result33'></p></div>
<div>
    <p id='result34'></p></div>
<div>
    <p id='result35'></p></div>
<div>
    <p id='result36'></p></div>
<div>
    <p id='result37'></p></div>
<div>
    <p id='result38'></p></div>
<div>
    <p id='result39'></p></div>
<div>
    <p id='result40'></p></div>
<div>
    <p id='result41'></p></div>
<div>
    <p id='result42'></p></div>
<div>
    <p id='result43'></p></div>
<div>
    <p id='result44'></p></div>
<div>
    <p id='result45'></p></div>
<div>
    <p id='result46'></p></div>
<div>
    <p id='result47'></p></div>
<div>
    <p id='result48'></p></div>
<div>
    <p id='result49'></p></div>
<div>
    <p id='result50'></p></div>
<div>
    <p id='result51'></p></div>
<div>
    <p id='result52'></p></div>
```

```
<div>
    <p id='result53'></p></div>
<div>
    <p id='result54'></p></div>
<div>
    <p id='result55'></p></div>
<div>
    <p id='result56'></p></div>
<div>
    <p id='result57'></p></div>
<div>
    <p id='result58'></p></div>
<div>
    <p id='result59'></p></div>
<div>
    <p id='result60'></p></div>
<div>
    <p id='result61'></p></div>

    </div>

    <script src="http://www.phage-therapy.org/scripts/xresistance_avoider.js"></script>


</P>


</P><P id="Additional"><BR><BR>

<iframe src="http://www.facebook.com/plugins/like.php?href=http://www.phage-therapy.org/calculators/xre-
sistance_avoider.html"
        scrolling="no" frameborder="0"
        style="border:none; width:450px; height:24px"></iframe>

</P>

<div id="googlesearchpositionalt">

<script>
  (function() {
    var cx = '018033303197021436553:1odoahemmfs';
    var gcse = document.createElement('script');
```

```
    gcse.type = 'text/javascript';
    gcse.async = true;
    gcse.src = 'https://cse.google.com/cse.js?cx=' + cx;
    var s = document.getElementsByTagName('script')[0];
    s.parentNode.insertBefore(gcse, s);
  })();
</script>
<gcse:search></gcse:search>

</div>

<script type="text/javascript"> other_links("BX","Cross Resistance Avoider","F","F","F","F","BEG","F");
</script>

</P>

<script type="text/javascript" src="http://www.biologyaspoetry.com/scripts/end_materials.js"> </script>

</body>
</html>
```

S5. www.phage-therapy.org/scripts/xresistance_avoider.js [return to Table of Contents]

```
// the function which handles the input field logic
function cocktail_optimizer() {

    var start = new Date();

    const maxphagenum = 100;
    const maxbacterianum = 500;
    const maxsizecoctail = 20;
    const maxtodisplay = 50;
    const numberofhitsconst = 2;  // zero for host-range breadth analysis

    var numbersofphagesField1 = document.getElementById('numbersofphagesField1').value;
    var numbersofphagesField2 = document.getElementById('numbersofphagesField2').value;
    var numbersofphagesField3 = document.getElementById('numbersofphagesField3').value;
    var numbersofphagesField4 = document.getElementById('numbersofphagesField4').value;

    var numbersofbacteriaField = document.getElementById('numbersofbacteriaField').value;

    var TwoDarrayField1 = document.getElementById('TwoDarrayField1').value;
    var TwoDarrayField2 = document.getElementById('TwoDarrayField2').value;
    var TwoDarrayField3 = document.getElementById('TwoDarrayField3').value;
    var TwoDarrayField4 = document.getElementById('TwoDarrayField4').value;

// To delete:    var numberofhitsField = document.getElementById('numberofhitsField').value;
// To delete:    var maxsizecocktailField = document.getElementById('maxsizecocktailField').value;
// To delete:    var maxnumbershownField = document.getElementById('maxnumbershownField').value;

    var deptharr = new Array(numbersofbacteriaField);

    var errorvar = 'F';
    var i = 0;
    var j = 0;
    var k = 0;
//    var teststring = '';

    result0.textContent = '----------------------------------------------------------------------';

//////////////////////////////////////////////////////////////////////////////
// INPUT ERROR CHECKING
```

```
    if (numbersofphagesField1.length < 1 || numbersofphagesField1 <= 2 || numbersofphagesField1 > maxphagenum
|| numbersofphagesField2.length < 1 || numbersofphagesField2 <= 2 || numbersofphagesField2 > maxphagenum) {

    errorvar = 'T';
    result1.textContent = 'number of phages must be > 2 and < ' + (maxphagenum+1);
    result2.textContent = '----------------------------------------------------------------------';

    }

// NOTE ABOVE THAT numbersofphagesField3 OR numbersofphagesField4 IS NOT BEING ERROR CHECKED!

    if ((numbersofbacteriaField.length < 1 || numbersofbacteriaField <= 2 || numbersofbacteriaField > maxbac-
terianum) && (errorvar == 'F')) {

    errorvar = 'T';
    result1.textContent = 'number of bacteria must be > 2 and < ' + (maxbacterianum+1);
    result2.textContent = '----------------------------------------------------------------------';

    }

// To delete:    if ((maxsizecocktailField.length < 1 || maxsizecocktailField <= 1 || maxsizecocktailField >
maxsizecoctail) && (errorvar == 'F')) {
// To delete:
// To delete:    errorvar = 'T';
// To delete:    result1.textContent = 'proposed cocktails must be > 1 but < ' + maxsizecoctail + ' (sorry)';
// To delete:    result2.textContent = '----------------------------------------------------------------------
';
// To delete:
// To delete:    }
// To delete:
// To delete:    if ((maxsizecocktailField.length < 1 || maxsizecocktailField > numbersofphagesField1 - 2) &&
(errorvar == 'F')) {
// To delete:
// To delete:    errorvar = 'T';
// To delete:    result1.textContent = 'cocktail size must be < number of phages tested - 1';
// To delete:    result2.textContent = '----------------------------------------------------------------------
';
// To delete:
// To delete:    }
// To delete:
// To delete:    if ((numberofhitsField.length < 1 || numberofhitsField < 1 || numberofhitsField >
maxsizecocktailField) && (errorvar == 'F')) {
```

```
// To delete:
// To delete:    errorvar = 'T';
// To delete:    result1.textContent = 'Each bacterium \'hit\' by < 1 or > cocktail size phages';
// To delete:    result2.textContent = '-----------------------------------------------------------------
';
// To delete:
// To delete:    }
// To delete:    else
// To delete:    {
// To delete:    numberofhitsField = numberofhitsField - 1;
// To delete:    }
// To delete:
// To delete:
// To delete:    if ((maxnumbershownField.length < 1 || maxnumbershownField <= 2 || maxnumbershownField >
maxtodisplay) && (errorvar == 'F')) {
// To delete:
// To delete:    errorvar = 'T';
// To delete:    result1.textContent = 'only the top ' + maxtodisplay + ' results will be shown (sorry)';
// To delete:    result2.textContent = '-----------------------------------------------------------------
';
// To delete:
// To delete:    }
// To delete:

    if ((TwoDarrayField1.length < 1 || TwoDarrayField2.length) < 1 && errorvar == 'F') {
        result1.textContent = 'you must enter 2 2D arrays to run this app';
        result2.textContent = '-----------------------------------------------------------------------';
        errorvar = 'T';
        }
    else {
        var numberrows = new Array(numbersofbacteriaField);
        for (i = 0; i < numberrows.length; i++) {
            numberrows[i] = 0;
        }

//    var bacteriaarray = new Array(numbersofphagesField1);
//    for (j = 0; j < bacteriaarray.length; j++) {
//        bacteriaarray[j] = 0;
//    }
//
//    bacteriaarrayinit = bacteriaarray;
```

```
    var TwoDArray1 = new Array(numbersofbacteriaField);
    var TwoDArray2 = new Array(numbersofbacteriaField);

    for (k = 0; k < numbersofbacteriaField; k++) {
        TwoDArray1[k] = new Array(numbersofphagesField1);
        TwoDArray2[k] = new Array(numbersofphagesField2);
    }

    for (k = 0; k < TwoDArray1.length; k++) {
        for (j = 0; j < numbersofphagesField1; j++) {
            TwoDArray1[j] = 0;
    } }

    for (k = 0; k < TwoDArray2.length; k++) {
        for (j = 0; j < numbersofphagesField2; j++) {
            TwoDArray2[j] = 0;
    } }

    for (k = 0; k < numbersofbacteriaField; k++) {
        deptharr[k] = -1;
    }

    result1.textContent = '';
    result2.textContent = '';
    result3.textContent = '';
    result4.textContent = '';
    result5.textContent = '';
    result6.textContent = '';

if (TwoDarrayField1.length > 0) {
    j = 0;
    m = 0;
    for (i = 0; i < TwoDarrayField1.length; i++) {
        m = m + 1;
        if (TwoDarrayField1.charCodeAt(i) == 10) {
            j = j + 1
            if (m-1 != numbersofphagesField1) {
                result1.textContent = 'Group 1 has ' + (m-1) + ' phages in a row rather than ' + number-
sofphagesField1;
                errorvar = 'T';
                }
            m = 0;
```

```
            }
        if (TwoDarrayField1.charCodeAt(i) != 10 && TwoDarrayField1.charCodeAt(i) != 48 && TwoDarray-
Field1.charCodeAt(i) != 49) {
            result1.textContent = 'An illegal character ("' + TwoDarrayField1[i] + '") is found in group 1';
            errorvar = 'T';
            }
        }
        if (m != numbersofphagesField1) {
            result1.textContent = 'Group 1 has ' + (m) + ' phages in a row rather than ' + numbersofphag-
esField1;
            if (m == 0) {
                result2.textContent = 'This could be a carriage return hanging off the end';
                }

            errorvar = 'T';
            }
}

if (TwoDarrayField2.length > 0) {
    k = 0;
    m = 0;
    for (i = 0; i < TwoDarrayField2.length; i++) {
        m = m + 1;
        if (TwoDarrayField2.charCodeAt(i) == 10) {
            k = k + 1
            if (m-1 != numbersofphagesField2) {
                result1.textContent = 'Group 2 has ' + (m-1) + ' phages in a row rather than ' + number-
sofphagesField2;
                errorvar = 'T';
                }
            m = 0;
            }
        if (TwoDarrayField2.charCodeAt(i) != 10 && TwoDarrayField2.charCodeAt(i) != 48 && TwoDarray-
Field2.charCodeAt(i) != 49) {
            result1.textContent = 'An illegal character ("' + TwoDarrayField2[i] + '") is found in group 2';
            errorvar = 'T';
            }
        }
        if (m != numbersofphagesField2) {
            result1.textContent = 'Group 2 has ' + (m) + ' phages in a row rather than ' + numbersofphag-
esField2;
            if (m == 0) {
```

```
                result2.textContent = 'This could be a carriage return hanging off the end';
                }
            errorvar = 'T';
            }
}

if (TwoDarrayField3.length > 0) {
    L = 0;
    m = 0;
    for (i = 0; i < TwoDarrayField3.length; i++) {
        m = m + 1;
        if (TwoDarrayField3.charCodeAt(i) == 10) {
            L = L + 1
            if (m-1 != numbersofphagesField3) {
                result1.textContent = 'Group 3 has ' + (m-1) + ' phages in a row rather than ' + number-
sofphagesField3;
                errorvar = 'T';
                }
            m = 0;
            }
        if (TwoDarrayField3.charCodeAt(i) != 10 && TwoDarrayField3.charCodeAt(i) != 48 && TwoDarray-
Field3.charCodeAt(i) != 49) {
            result1.textContent = 'An illegal character ("' + TwoDarrayField3[i] + '") is found in group 3';
            errorvar = 'T';
            }
        }
        if (m != numbersofphagesField3) {
            result1.textContent = 'Group 3 has ' + (m) + ' phages in a row rather than ' + numbersofphag-
esField3;
            if (m == 0) {
                result2.textContent = 'This could be a carriage return hanging off the end';
                }
            errorvar = 'T';
            }
}


if (TwoDarrayField4.length > 0) {
    Q = 0;
    m = 0;
    for (i = 0; i < TwoDarrayField4.length; i++) {
        m = m + 1;
```

```
        if (TwoDarrayField4.charCodeAt(i) == 10) {
            Q = Q + 1
            if (m-1 != numbersofphagesField4) {
                result1.textContent = 'Group 4 has ' + (m-1) + ' phages in a row rather than ' + number-
sofphagesField4;
                errorvar = 'T';
                }
            m = 0;
            }
        if (TwoDarrayField4.charCodeAt(i) != 10 && TwoDarrayField4.charCodeAt(i) != 48 && TwoDarray-
Field4.charCodeAt(i) != 49) {
            result1.textContent = 'An illegal character ("' + TwoDarrayField4[i] + '") is found in group 4';
            errorvar = 'T';
            }
        }
        if (m != numbersofphagesField4) {
            result1.textContent = 'Group 4 has ' + (m) + ' phages in a row rather than ' + numbersofphag-
esField4;
            if (m == 0) {
                result2.textContent = 'This could be a carriage return hanging off the end';
                }
            errorvar = 'T';
            }
}


    if (errorvar == 'F') {
        if (numbersofphagesField1 == numbersofphagesField2) {
            if (TwoDarrayField1.length > TwoDarrayField2.length) {
                result1.textContent = 'Group #2 is missing data relative to Group #1';
                errorvar = 'T';
                }
            if (TwoDarrayField2.length > TwoDarrayField1.length) {
                result1.textContent = 'Group #1 is missing data relative to Group #2';
                errorvar = 'T';
                }
            }
        if (TwoDarrayField3.length > 0) {
            if (numbersofphagesField1 == numbersofphagesField3) {
                if (TwoDarrayField1.length > TwoDarrayField3.length) {
                    result1.textContent = 'Group #3 is missing data relative to Group #1';
                    errorvar = 'T';
```

```
                    }
                if (TwoDarrayField3.length > TwoDarrayField1.length) {
                    result1.textContent = 'Group #1 is missing data relative to Group #3';
                    errorvar = 'T';
                    }
                }
            if (numbersofphagesField2 == numbersofphagesField3) {
                if (TwoDarrayField2.length > TwoDarrayField3.length) {
                    result1.textContent = 'Group #3 is missing data relative to Group #2';
                    errorvar = 'T';
                    }
                if (TwoDarrayField3.length > TwoDarrayField2.length) {
                    result1.textContent = 'Group #2 is missing data relative to Group #3';
                    errorvar = 'T';
                    }
                }
            }

        if (numbersofbacteriaField != j+1) {
            result1.textContent = '# of bacteria being tested (' + numbersofbacteriaField + ') must = # rows
(' + (j+1) + ' @ 1)';
            result2.textContent = 'or you might have a carriage return hanging off end';
            errorvar = 'T';
            }
        if (numbersofbacteriaField != k+1) {
            result1.textContent = '# of bacteria being tested (' + numbersofbacteriaField + ') must = # rows
(' + (k+1) + ' @ 2)';
            result2.textContent = 'or you might have a carriage return hanging off end';
            errorvar = 'T';
            }
        }


// If we've made it this far then that means that the number of rows equals the number of bacteria indicated
and this needs to be truy for both arrays.

    i = 0;
    j = 0;
    k = 0;
    L = 0;
    Q = 0;
    m = 0;
```

```
    n = 0;
    w = parseInt(numbersofbacteriaField,10);
    x = parseInt(numbersofphagesField1,10);
    y = parseInt(numbersofphagesField2,10);
    z = parseInt(numbersofphagesField3,10);
    zz = parseInt(numbersofphagesField4,10);
    max = 0;
    localmax = 0;
    onetracker = 0;
    twotracker = 0;
    threetracker = 0;
    fourtracker = 0;
    counttracker = 0;

    for (i = 0; i < numbersofbacteriaField; i++) {
        max = 0;
        for (j = 0; j < x; j++) {
            for (k = 0; k < y; k++) {
                for (L = 0; L < z; L++) {
                    for (Q = 0; Q < zz; Q++) {
                        localmax = parseInt(TwoDarrayField1[(i*(x+1))+j],10) + parseInt(TwoDarray-
Field2[(i*(y+1))+k],10);
                        if (TwoDarrayField3.length > 0) {
                            localmax = localmax + parseInt(TwoDarrayField3[(i*(z+1))+L],10);
                            }
                        if (TwoDarrayField4.length > 0) {
                            localmax = localmax + parseInt(TwoDarrayField4[(i*(zz+1))+Q],10);
                            }
                        if (localmax > max) {
                            max = localmax;
                            }
                        }
                    }
                }
            }
        if (max > 0) {
            onetracker = onetracker + 1;
            }
        if (max > 1) {
            twotracker = twotracker + 1;
            }
        if (max > 2) {
```

```
            threetracker = threetracker + 1;
            }
        if (max > 3) {
            fourtracker = fourtracker + 1;
            }
        counttracker = counttracker + 1;
        deptharr[i] = max;
        }

    if (errorvar != 'T') {
    result1.textContent = 'Depth per bacterium (rows transposed to horizontal):'
    result2.textContent = deptharr.toString();
    result3.textContent = 'This is ">0" depth with ' + (Math.round(10000*onetracker/counttracker)/100) + '%
breadth (' + onetracker + '/' + counttracker + ').';
    result4.textContent = 'This is ">1" depth with ' + (Math.round(10000*twotracker/counttracker)/100) + '%
breadth (' + twotracker + '/' + counttracker + ').';
    result5.textContent = 'This is ">2" depth with ' + (Math.round(10000*threetracker/counttracker)/100) + '%
breadth (' + threetracker + '/' + counttracker + ').';
    result6.textContent = 'This is ">3" depth with ' + (Math.round(10000*fourtracker/counttracker)/100) + '%
breadth (' + fourtracker + '/' + counttracker + ').';
       }
    result7.textContent = '---------------------------------------------------------------------';

// :-) to here for Groups 1 and 2! (except still need to remove a lot of code above...)

    }

}

// use an eventlistener for the event
var subButton1 = document.getElementById('subButton1');
subButton1.addEventListener('click',cocktail_optimizer, false);
```
